# Morphological Characterization of High Molecular Weight Poly(styrene-*b*-isoprene) or PS-*b*-PI and Its Hydrogenated, Sulfonated Derivatives: An AFM Study

**DOI:** 10.3390/polym17223047

**Published:** 2025-11-17

**Authors:** Nikolaos Politakos, Galder Kortaberria, Apostolos Avgeropoulos

**Affiliations:** 1 Institute of Chemical Biology, National Hellenic Research Foundation, 11635 Athens, Greece; 2 Department of Chemical and Environmental Engineering, University of the Basque Country (UPV/EHU), 20018 Donostia, Spain; galder.cortaberria@ehu.eus; 3 Department of Materials Science Engineering, University of Ioannina, 45110 Ioannina, Greece

**Keywords:** hydrogenation, sulfonation, poly(isoprene), anionic polymerization, atomic force microscopy (AFM), self-assembly, nanostructures, chemical modification reactions, molecular characterization, thin films

## Abstract

The surface morphology of high molecular weight poly(styrene-*b*-isoprene) block copolymer was analyzed after chemical modification. Poly(styrene-*b*-isoprene) was converted into poly(styrene-*b*-(ethylene-alt-propylene)) by hydrogenation and into poly(styrene-*b*-sulfonated isoprene) by mild sulfonation of the PI block. Obtained morphologies were examined by atomic force microscopy, analyzing the effect of sample preparation parameters such as solvent (tetrahydrofuran, toluene, and cyclohexane), casting technique (spin casting and drop casting), and annealing temperature [room temperature, 80, 100, and 120 °C]. Significant morphological and topographical changes were found depending on the different parameters. Each modification step introduces new variables that can affect the final structure and properties of the copolymer. Finding the balance between solvent choice, casting technique, and annealing conditions was a difficult task and required extensive experimentation and understanding of the principles of block copolymer self-assembly.

## 1. Introduction

New challenges in nanotechnology lead to the demand of innovative materials for different applications. Using novel polymeric systems with new monomers and different architectures is one way to obtain the necessary materials. Polymers can be synthesized with different methods, achieving different architectures and block combinations [1,2,3,4,5,6,7,8,9,10]. The capability of block copolymers to self-assemble into different nanostructures justifies their suitability for use in advanced technologies [1,2,3,11,12,13]. Self-assembly is fast, easily scalable, and relatively cheap, and from a technological perspective, provides a pathway to synthesize materials from the nm to μm range [1,2]. Depending on the morphologies (shape, size, and periodicity), it can be suitable for numerous applications such as drug delivery [14], electronics [15], masks [2,16,17], nanopatterning [3,18,19,20], functional nanomaterials [3,21,22], and nanoporous membranes [3,23,24,25,26], among others. Block copolymers can also be combined with other materials, such as ceramic or metallic nanoparticles [3,27,28,29,30,31,32], leading to new physical, chemical, magnetic, or electric properties [3].

The basic key for self-assembly into ordered nanostructures is based on the blocks’ thermodynamic incompatibility. Different morphologies can be obtained depending on the blocks’ volume fraction, the variability of the Flory–Huggins interaction parameter (χ), the polymerization degree (N), and sample preparation parameters such as solvent or annealing temperature. The system will evolve to different morphologies to minimize free energy [27,33].

Selective chemical modification of one block by mild reactions is a beneficial technique to obtain new copolymers. A copolymer can be altered into a new material with different physical and chemical properties. It becomes essential when the polymerization of new monomers is complex, very difficult with low yields, or needs special conditions. By changing the chemical structure, the Flory–Huggins (χ) parameter is also altered, thus leading to nanostructures that can vary from the initial copolymer. Polydienes [especially poly(butadiene) and poly(isoprene)] are very suitable for chemical modification due to the existing double bond per monomeric unit [34]. Chemical modification can also be used for preparing amphiphilic copolymers, transforming one of the blocks into hydrophilic (PS modification with sulfonation, for example), which may result in interesting properties with a variety of applications, such as in micelles, polymeric surfactants, emulsifiers, foam, and stabilizers [35,36,37,38].

Atomic force microscopy (AFM) is a powerful technique for analyzing the surfaces of various samples and understanding their properties. It is a method which is continuously evolving, introducing new features and expanding its capabilities. AFM probes are becoming increasingly sophisticated, allowing for applications in the mechanical and electrical characterization of samples, as well as measurements in liquid environments and even within living cells [39]. AFM offers exciting potentials for cutting-edge applications, including the measurement of surface tension [40], adhesion [41], and the mechanical properties of soft tissues [42,43]. It can also be used for direct measurement of nanoindentation [44], investigation of magnetic properties [45], assessment of electrical conductivity [46], and analysis of the morphology and micromechanical properties of the membranes of specific human cells [47]. It is far from certain that AFM can provide micromechanical and physical characterization of various objects, such as red blood cells, with force-sensing capabilities to measure properties like surface topography and stiffness at the nanoscale [48].

**Scheme 1:**
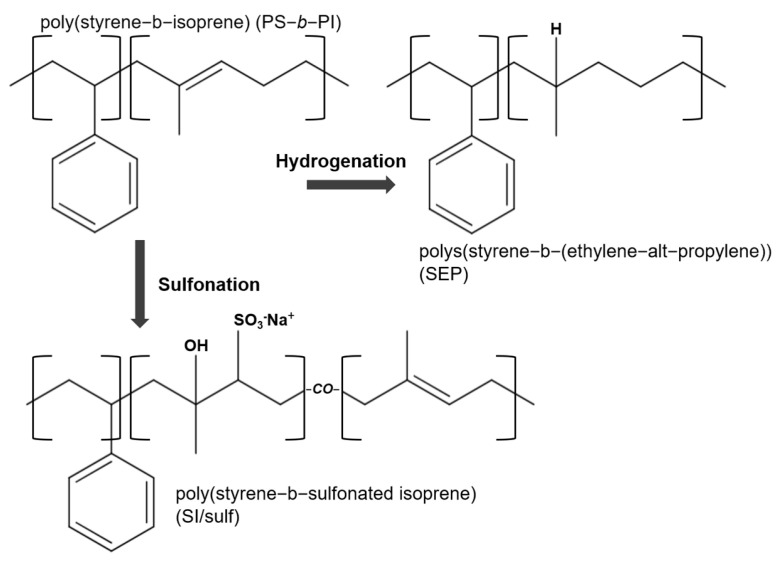
Chemical structures of the poly(styrene-*b*-isoprene) (PS-*b*-PI or SI) diblock copolymer before and after chemical modification exclusively of the PI segments by hydrogenation leading to poly(styrene-*b*-(ethylene-alt-propylene)) (SEP) and by sulfonation obtaining poly(styrene-*b*-sulfonated isoprene) (SI/sulf).

This manuscript aims to analyze the surface morphology of a high molecular weight poly(styrene-*b*-isoprene) (PS-*b*-PI or SI) diblock copolymer before and after chemical modification exclusively of the PI segments by hydrogenation leading to poly(styrene-*b*-(ethylene-alt-propylene)) (PS-*b*-P(E-alt-P)) or SEP) and by sulfonation obtaining poly(styrene-*b*-sulfonated isoprene) (PS-*b*-(PI-r-PIs)) or SI/sulf). In Scheme 1 the chemical structures of the pristine diblock copolymer and the two modified cases are evident. The latter chemical modification does not yield to 100% modified monomeric units (42% is sulfonated PI). Besides the effect of chemical modification on the resulting morphologies, the sample preparation parameters are also discussed, such as solvent (toluene, tetrahydrofuran, and cyclohexane), film preparation technique (spin and drop casting), and annealing temperature (room temperature (R.T.), 80°, 100°, and 120 °C). Different chemical structures are sensitive to selective or nonselective solvents, while after modification, the nature of PI and the χ parameter between PI and PS changes, leading to different morphologies, also affected by annealing treatment and film preparation techniques. This manuscript is an add-on study to a previous work in which a PS-*b*-PI diblock copolymer was studied but with lower overall number average molecular weight [49]. The molecular weight parameter is important when it is considered in the context of microphase separation (degree of polymerization, N, is directly related to molecular weight and is an important variable for microphase separation fully dependent on the χN value). As molecular weight increases, χN increases, driving the system further into the microphase separation regime. The same copolymer and its lower-molecular-weight analogous may not fully organize, whereas the higher-molecular-weight analogous does, due to the increased χN value.

In this study, the initial diblock copolymer has similar volume fractions for both blocks and a higher molecular weight than in our previous study. Finally, the molecular weight is also essential for determining the size of the microphase-separated domains, such as lamellae or cylinders. The longer chains must stretch farther to segregate into their respective domains, increasing the system’s energy. To minimize this energy, the polymers form larger domains, which allows the chains to adopt less-stretched conformations.

## 2. Materials and Methods

### 2.1. Materials

The PS-*b*-PI (SI) diblock copolymer used in this work was synthesized by sequential anionic polymerization using high vacuum techniques. The number average molecular weight of the diblock copolymer was 187,000 g/mol, dispersity (Đ) lower than 1.1, with 53% being PS and 47% of PI. Purification of all chemical reagents (styrene and isoprene from Acros, Geel, Antwerpen Belgium and benzene from Lab-scan, Bangkok, Thailand) to the standards required for anionic polymerization has been described elsewhere [50]. Hydrogenation and sulfonation, both by mild reactions, were carried out as mentioned in the literature [49]. p-Toluenesulfonyl hydrazide (Sigma-Aldrich, St. Louis, MO, USA, 97%) was used for hydrogenation with p-xylene (Fluka, Pensacola, FL, USA, ≥99.5%) as solvent. Anhydrous sulfuric acid (Panreac, Barcelona, Spain, 96%) with 1,4-dioxane (Sigma-Aldrich, ≥99%) as solvent, methanol (Lab-scan, Puchheim, Germany, 99.8%), and 15 wt% aqueous solution of NaOH were used also in the sulfonation procedure. Finally, tetrahydrofuran (THF) (Lab-scan, 99.8%), toluene (Lab-scan, 99.8%), and cyclohexane (Panreac, 99.5%) were used as solvents for film preparation.

### 2.2. Characterization

Morphological characterization was conducted by atomic force microscopy (AFM). Thin films were prepared by casting or spin casting of 3 wt% solutions of copolymers in different solvents onto glass substrates. Spin-coating was conducted at 2000 rpm for 120 s using a Telstar Instrument P-6708D spin-coater(Terrassa, Barcelona, Spain). After complete evaporation of the solvent, films were then annealed in a high-vacuum oven (Binder, Tuttlingen, Germany) for 24 h at different temperatures. A Dimension 3100/Nanoscope IVA, Veeco, New York, NY, USA (from Digital Instruments Santa Barbara, CA, USA) was used. Tapping mode in air was employed using a TESP-V2 probe made from monolithic silicon which is highly doped to dissipate static charge, chemically inert, and offers a high mechanical Q-factor for high sensitivity. The AFM tip is uncoated and shaped like a polygon-based pyramid. The shape of the tip is standard with a radius of <8 nm (<12 nm guaranteed) and with cantilever shaped as beam with force constant 42 N/m, resonance frequency 320 kHz, length 125 µm, width 30 µm, and thickness 4 µm, at a scan rate of 1.0 Hz and a resonance frequency of ~300 kHz. Measurements were performed with 512 scan lines. The images presented here were scanned at a scale bar of 3 μm × 3 μm and a 3D representation was reconstructed from the same image at a scale bar of 1 μm × 1 μm. The profile of the mean value of the phase was also extracted at a diagonal of the initial image at 1.5 μm in the X axis with the Y axis representing the phase. For the measurements of the mean roughness S_a_, three distinct areas of 3 μm × 0.5 μm of the initial image were selected and the mean value was presented. From the same areas, the mean value for S_k_ (skewness) was also presented. The skewness is presented only for the samples that had some type of morphology. The skewness will show a dominance of high peaks when it is positive or dominance of valleys when it is negative. In the case of 0 or close to 0, a symmetrical existence of both peaks and valleys is present. In the same images, the sizes of characteristic domains were also calculated by averaging 3 to 5 points. For the analysis of the AFM images, Gwyddion 2.69 (released 28 July 2025) (https://gwyddion.net/) was used.

Proton nuclear magnetic resonance spectroscopy (^1^H-NMR) was used for the verification of the sulfonation. The sample was dissolved in deuterated chloroform (CDCl_3_). The spectra were recorded at room temperature (R.T.) on an Avance Bruker 500 MHz (Rheinstetten, Germany) equipped with BBO z-gradient probe Bruker DSX NMR spectrometer (Billerica, MA, USA) using a rate of 5000 Hz, a frequency of 500 MHz, and a delay of 1 s.

For the Fourier-transform infrared spectroscopy (FTIR) and size exclusion chromatography (SEC) instruments, the experimental conditions can be found in the Supporting Information.

## 3. Results

### 3.1. Molecular Characterization

The molecular characterization instrumentation was thoroughly mentioned and analyzed for derivatives diblock copolymers in a previous work [49]. Size exclusion chromatography (SEC) was conducted to verify that no side reactions occurred during the synthesis of the diblock copolymer. Furthermore, Fourier transform infrared spectroscopy (FTIR), and proton nuclear magnetic resonances (^1^H-NMR) were conducted to verify the success of the chemical modification reactions. Hydrogenation and sulfonation yields of 100% and 42% were obtained, respectively. Thus, in the SI/sulf copolymer, sulfonated and non-sulfonated PI blocks are present randomly located throughout the PI segments. The ^1^H-NMR spectra for the result of the sulfonation reaction is shown in Figure S1 (Supporting Information).

### 3.2. Morphological Characterization

Solubility parameters for different solvents and blocks as well as polymer–polymer (χ_AΒ_) and polymer–solvent (χ_SP_) interaction parameters are given in Table 1 and Table 2, respectively, together with the necessary equations needed for their calculation [51,52].

The affinity between polymer–polymer and polymer–solvent is an essential parameter to explain the observed morphologies. The affinity of blocks with solvent molecules will affect their position and orientation onto the substrate after solvent evaporation. A solvent with high affinity will move the block towards the interface with air, while the block will be located at the substrate interface with a non-miscible solvent [55]. As evident by the calculated interaction parameter values, THF is a selective solvent for PS and PI/sulf (much lower affinity with non-polar toluene and cyclohexane) blocks. However, its affinity is higher with PS. On the other hand, toluene is a selective solvent for PI and PEP (lower affinity than PI); in this case, affinity with cyclohexane and THF is slightly lower but quite similar.

Regarding interaction parameters among the blocks, PS and PI present the highest affinity. PS and PI are supposed to present higher miscibility than PS and PEP so that a higher organized nanostructure can be expected for the SEP copolymer than for the initial diblock copolymer precursor. The SI/sulf case is more complicated since it contains three different blocks: PS (~50 wt%), PI (~30 wt%), and PI/sulf (~20 wt%). The overall morphology can be expected to be affected by more parameters than the other copolymers. As PI/sulf presents low affinity with PS and PI (1.35 and 1.93, respectively), immiscibility among the blocks is expected, leading to nanostructured morphologies due to chemical incompatibility among the segments [56]. Depending on the annealing treatment, different nanostructures are expected according to the literature [57].

**Figure 1:**
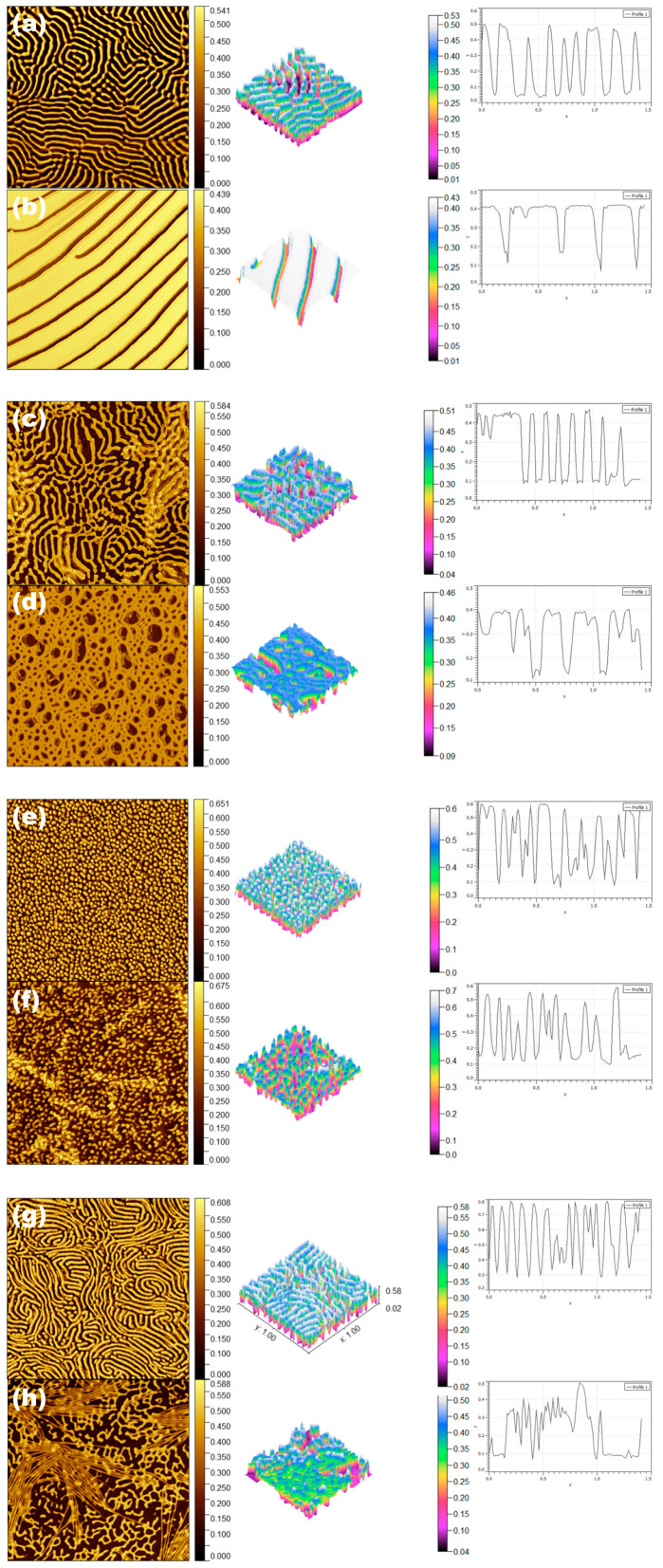
Examples of all morphologies observed by AFM phase images (3 μm × 3 μm) with a 3D representation of a 1 μm × 1 μm and a profile of the morphology of 1.5 μm diagonal: (**a**) lamellae (SEP, spin casted from toluene annealed at R.T.), (**b**) not fully organized lamellae (SI, casted from toluene annealed at R.T.), (**c**) lamellae not fully organized lamellae with crystallites (SEP, spin casted from toluene annealed at 100 °C), (**d**) polymeric network (SEP, casted from cyclohexane annealed at 120 °C), (**e**) spherical micelles (SEP, casted from cyclohexane annealed at R.T.), (**f**) spherical micelles with crystallites (SI/sulf, spin casted from cyclohexane annealed at 80 °C), (**g**) worm-like (SI, casted from cyclohexane annealed at R.T.) and (**h**) worm-like with crystallites (SEP, spin casted from THF annealed at 100 °C).

The morphology of the initial SI copolymer is affected when chemical modification reactions occur. After complete hydrogenation of the PI, a new PEP block is generated (showcasing variable affinities with blocks and solvents), and therefore different morphologies are expected from those of the initial SI. In the same manner, after the sulfonation reaction, different topological structures are observed for the PI, PI/sulf, and PS blocks. Table 3 shows the morphologies obtained for all copolymers under different conditions. Figure 1 gives examples of all kinds of morphologies described in Table 3. In Figure S2 (Supporting Information), the phase images for all samples are presented with a 3D representation and a cut-off profile.

In the phase images, it is evident that stiffer materials appear brighter, while the softer phases of the copolymers appear darker. This contrast highlights the distinct characteristics of each material, emphasizing their varying mechanical properties. Figure 1 clearly demonstrates that the polystyrene phase exhibits greater stiffness compared to the polydienic segments, as seen in Figure 1b and 1g, respectively. Furthermore, in the images featuring the hydrogenated sample (Figure 1c–e,h), the PS segment again stands out as brighter.

Table 3 presents findings related to how various factors such as solvent type, annealing temperature, and casting technique influence the resulting morphologies of copolymer samples. Notably, disordered morphologies are predominantly observed in spin-casted samples that are subjected to high annealing temperatures of 120 °C across all solvent types used. This phenomenon is attributed to the rapid evaporation of the solvent, which does not allow adequate time for the molecules to organize themselves into distinct and well-defined nanostructures, as supported by prior research [58]. To achieve ordered or partially ordered morphologies, specific parameters must be optimized. The casting technique has a considerable impact, resulting in alternating lamellae structures, particularly when using toluene and THF at an annealing temperature of 80 °C. In contrast, the simple casting method tends to favor the formation of less ordered lamellae structures, especially for samples that are annealed at R.T. Furthermore, less organized lamellae containing crystallites are detected in samples that have been spin cast from toluene, suggesting that the solvent plays a role in determining the degree of order in the film structure. Network-type topologies are primarily observed in samples that have been spin cast from all solvent types and subsequently annealed at the high temperature of 120 °C.

In samples prepared with cyclohexane, spherical micelles become pronounced, particularly for those that are annealed from R.T. up to 100 °C. Notably, in spin-casted SI/sulf samples, crystallites are observed in conjunction with these spherical micelles. Worm-like structures are predominantly found in samples spin cast from THF, with the specific morphology depending on the copolymer under consideration. For example, the SEP sample spin cast with THF and annealed at 100 °C displays both crystallite formation and a worm-like structure. The fast evaporation rate of THF is a critical factor, restricting the time available for the samples to develop fully ordered nanostructures, a trend consistent with other solvents as well. When analyzing the effects of solvents, it becomes apparent that cyclohexane typically results in the formation of spherical micelles and disordered structures. Films derived from toluene tend to exhibit more organized domains, primarily in the form of lamellae. Conversely, samples made from THF exhibit a range of morphologies, which may include either lamellae or worm-like structures, depending on the specific conditions applied during processing. This variability leads to many observed disordered morphologies due to THF’s high evaporation rate [59].

In terms of the effect of annealing temperature, the most well-ordered morphologies—primarily lamellae structures and spherical micelles—are most frequently identified at R.T. As the annealing temperature increases, a shift is noted towards more disordered structures alongside the simultaneous presence of ordered domains. This is primarily due to enhanced solubility at elevated temperatures, as indicated in previous studies [60]. The interplay between block miscibility and the thermal energy generated during annealing can inhibit the achievement of fully organized structures [61]. As the temperature rises, the increased thermal energy facilitates block mobility and rearrangement; however, the process of blocks migrating towards the surface complicates obtaining ideal morphologies. The influence of the casting technique is particularly noteworthy, as crystallites have only been identified in samples that were spin cast, underscoring the importance of this method in structuring the final films [62].

For copolymers, microphase separation is strongly linked to the glass transitions of both blocks, enabling the blocks to move to overcome thermodynamic barriers and form stable nanostructures. In addition to the temperature barrier, annealing time plays a crucial role in forming nanostructures. The polyisoprene and polyethylene/polypropylene show glass transitions below R.T., meaning that these blocks have high mobility; in contrast, the sulfonated polyisoprene has a significantly higher glass transition temperature but is still close to R.T. The different temperatures used in this study are capable of high mobility for all blocks except PS. The PS is close to the glass transition at 80 °C and 100 °C and has high mobility at 120 °C. The 100 °C barrier is the key to achieving full mobility of all chains across all blocks. The higher temperature of 120 °C leads the copolymer to form a disordered, single-phase melt, as seen in the majority of samples, which do not show ordered structures at this temperature.

When examining the morphologies corresponding to various copolymers, it is evident that the SI copolymer precursor tends to yield less organized structures compared to its hydrogenated counterpart, SEP. This difference is likely due to variations in block miscibility, with SI displaying a lower interaction parameter [63] than SEP, thus primarily resulting in disordered morphologies such as lamellae structures, spherical micelles, and worm-like forms, though these phenomena are only observable under specific processing conditions. In contrast, the SEP copolymer typically exhibits more ordered structures like lamellae—predicted due to the molecular weight ratio of the constituent blocks—and also features some spherical micelles and worm-like morphologies due to its higher interaction parameter between blocks. The case involving the SI/sulf copolymer is more intricate, given the presence of multiple components such as PI, PI/sulf, and PS, which leads to a complex interaction landscape between block/block and block/solvent. This amphiphilic nature of the copolymer is a significant contributor to the prevalent formation of spherical micelles in a majority of cases. Exceptions to this trend include the lamellae and network morphologies, particularly when utilizing toluene and THF as solvents.

In terms of skewness (Table S1, Supporting Information), most of the samples have values less than 1 (negative or positive), indicating a slight dominance of peaks or valleys, but are relatively symmetrical in the organized or almost organized domains. Notably, some samples also showed values very close to 0, indicating a quite symmetrical distribution of peaks and valleys. Finally, the majority of more asymmetrical morphologies are those in which the lamellas have not formed, showing negative values and the majority of valleys. Some other morphologies with negative skewness are also the SEP samples, cast from cyclohexane annealed at 80 and 120 °C (micelles and network), and SI/sulf, cast from THF at 100 degrees (micelles). The only positive skewness value above 1 is the sample SI/sulf spic cast from cyclohexane annealed at 100 °C (worm-like). The nature and evaporation rate of cyclohexane are helping to create these asymmetries.

Regarding the roughness of the samples, the unorganized samples have a smoother surface, because no structure forms. If one looks more closely at the organized domains and the nature of each sample, as well as the individual parameters such as solvent, casting technique, and temperature, some interesting points can be extracted. Among the samples, the rougher surfaces are found in the SEP samples, with an average roughness of 0.148, while SI has 0.111 and SI/sulf has 0.087. In terms of the solvents, the difference is not so crucial, with cyclohexane and THF showing slightly higher roughness (0.121 and 0.122), probably due to their higher evaporation rates and the time structures have to evolve. In the casting technique, a pattern is observed in the spin-cast samples, showing higher roughness (0.127) than in the casted ones (0.112). Here, the influence of the substrate is evident, as evidenced by the formation of thinner films in the spin-cast samples. For the different annealing temperatures, all show similar values, with 120 °C slightly higher. Finally, the different morphologies show a mixed trend in roughness values. The structures with lamellae morphology have an average value of 0.104, but if we look closer, the organized lamellae and the non-organized ones show a significant difference, with values of 0.130 vs. 0.087. This is mainly because the organized lamellae have defined the two polymeric components, and the stiffer PS is contributing 100%. The micellar structures have a low value of 0.107, indicating relatively smooth surfaces. Also important are the network and worm-like structures, which can be interpreted as intermediate structures with the polymeric components not fully organized, and this peculiar mixing shows roughness values of 0.146 (worm-like) and 0.141 (network).

The sizes of the different domains within the organized structures are also important points that must be addressed. For the micelles found for SEP, the sizes are very close, ranging from 73 nm to 80 nm. A similar trend was also observed for the micelles found for the SI/sulf. For the network morphologies of the SEP, the casting technique plays a key role, as in simple casting, the sizes are similar for PS and PEP at 64 and 63 nm, respectively. By introducing the spin-casting technique for the same sample, the PS domain maintains the same size, while the PEP increases to 179 nm. Finally, the solvent plays a role: the SEP sample has a similar size when cast from cyclohexane, whereas casting from THF results in a PEP size of 115 nm.

The worm-like morphologies also show interesting domain sizes, with the SI case ranging from 16 nm (cast from cyclohexane annealed at RT) to 71 nm (spin-cast from cyclohexane annealed at 80 °C). In the case of SEP, the PS has quite similar sizes, ranging from 41 to 54 nm (smaller than in the SI), whereas the PEP has a higher value, ranging from 55 to 82 nm. The trend, as mentioned above, is directly related to the organization of the PEP during casting and annealing, compared to the PI block found in SI systems. The worm-like morphologies in the SI/sulf do not show significant size changes, with PS ranging from 39 to 52 nm.

Finally, for the lamellae morphology, the only example organized for the SI system shows PS domains of 56 nm and PI of 27 nm, spin-cast from the annealed THF at 100 °C. Lamellae morphology for the SEP samples shows an impressive trend: samples cast and spin-cast from toluene annealed at RT show differences. In casting, the PS is 63 nm, while the PEP is 90 nm; in spin casting, the PS is reduced to 46 nm and the PEP to 79 nm, resulting in larger PEP domains. The effect of the solvent can be seen for the SEP system casted and annealed at 80 °C, where from toluene has sizes of PS 59 nm and PEP 81 nm, but changing the solvent, and thus the affinity of the blocks, the sizes are increasing to 81 nm for PS and 130 nm for PEP; a 37% increase for the PS and 60% increase for the PEP. Finally, increasing the annealing temperature to 120 °C increases the domain size for PS to 138 nm (compared to 59 nm at 80 °C) and for PEP to 119 nm (compared to 81 nm at 80 °C), increases of 134% and 47%, respectively. The 120 °C annealing temperature is crucial because it is above the glass transition temperature of PS, allowing sufficient mobility for organization. In the case of SI/sulf, the only sample with lamellae morphology (cast from THF at 80 °C) shows similar domain sizes of 82 nm and 79 nm.

**Figure 2:**
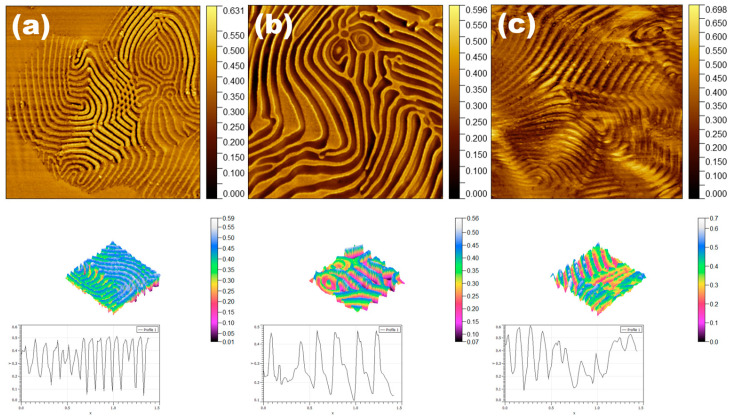
AFM phase images (3 μm × 3 μm) showing lamellae morphology observed for (**a**) SI, (**b**) SEP and (**c**) SI/sulf copolymers, showing phase and ordering differences. The 3D phase 1 μm × 1 μm and 1.5 diagonal profile sections are also shown.

Figure 2 compares lamellae morphologies obtained for SI, SEP, and SI/sulf copolymers. Figure 2a corresponds to SI copolymer spin cast from THF and annealed at 100 °C. As can be seen, the nanostructure is not fully ordered. The fast evaporation rate of THF and spin casting technique gave lamellas little time for complete formation [64]. Figure 2b corresponds to SEP copolymer cast from toluene and annealed at R.T. Due to the lower evaporation rate of solvent (together with the casting technique), lamellas can be fully formed, and they also show higher immiscibility among blocks for SEP when compared to SI.

Figure 2c shows lamellae morphology obtained for SI/sulf copolymer cast from THF and annealed at 80 °C. THF presents similar affinity with all blocks (χPS = 0.09, χPI = 0.16 and χPI/sulf = 0.34). With the casting technique, solvent evaporation is slower than by spin casting. Thus, llamelas have enough time to form fully.

The roughness of the different lamellae morphologies is obviously governed by the solvent, temperature, and casting technique, but it can be seen to be relatively smooth, with values of 0.064 to 0.094. The sizes of the PS domains are increasing from 56 nm at SI to 63 nm at SEP and 82 nm at SI/sulf. For the PI, the size is 27 nm; after modification, it increases to 90 nm at SEP and 79 nm for the sulfonated PI. The modification creates different balances among the PS chains, with the modified ones leading to changes in the sizes.

**Figure 3:**
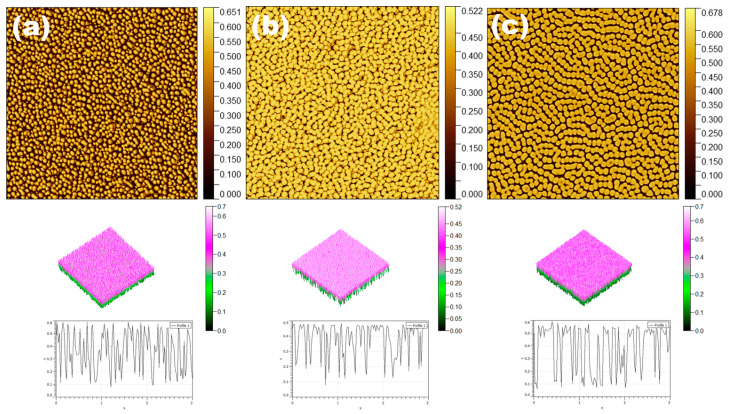
2D and 3D representation of (3 μm × 3 μm) for SEP copolymer casted from cyclohexane and annealed at different temperatures: (**a**) R.T., (**b**) 80 °C, and (**c**) 100 °C, showing differences in aggregation and organization of spherical micelles. Lower images correspond to 3 μm horizontal profile sections, showing phase differences.

The solvent, temperature, casting technique, and chemical nature of blocks also affect topography and the phase difference of morphologies [65]. The size, roughness, and distribution of lamellae, spherical micelle, and worm-like domains will change by altering the abovementioned parameters. As an example, in Figure 3, a 3D representation of the morphologies obtained can be observed for SEP copolymer as cast from cyclohexane and annealed at R.T., 80, and 100 °C, respectively. The interaction parameters of PS and PEP with cyclohexane are 0.36 and 0.16, respectively (as evident in Table 2).

PEP presents a higher affinity with cyclohexane than PS, and the solvent evaporates from the surface; therefore, the PS chains are removing the solvent faster than the PEP blocks. The solvent is maintained longer with PEP chains giving an extra volume and PS blocks tend to aggregate for minimizing energy. In that manner, PEP blocks create spherical micelles on the surface. Domain size and aggregation are controlled by annealing temperature. An estimation of size, roughness, and aggregation has been carried out for topography and phase by AFM. Dimensions for PS are 53, 73, and 80 nm, by annealing at R.T., 80, and 100 °C, respectively. At 100 °C, PS blocks could have enough energy to rearrange and create more stable domains, something that can also be seen from roughness (0.081), where at R.T. is rougher with a value of 0.157 and at 100 °C of 0.161. The 3D representation of the sample annealed at 80 °C has the most compact surface. The raise of the annealing temperature at 120 °C disrupts the morphology creating a network (Figure S2, Supporting Information).

**Figure 4:**
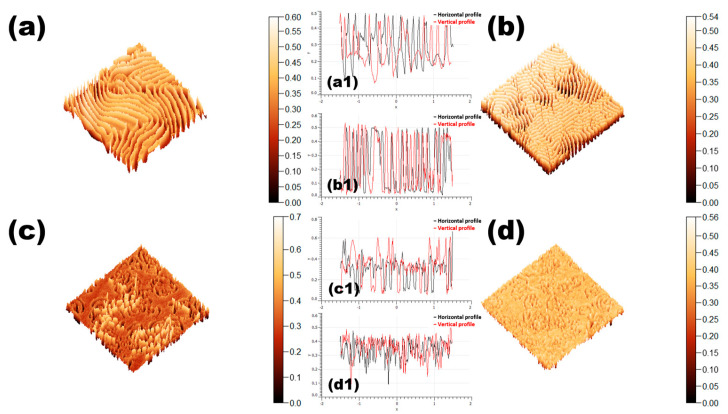
3D representation of AFM images (3 μm × 3 μm) for SEP copolymer: (**a**) casted and (**b**) spin casted from toluene and annealed at R.T. In (**c**,**d**), the 3D representation of a SI/sulf annealed at 80 °C spin cast from (**c**) toluene and (**d**) THF. Inner images (**a1**–**d1**) show the distribution of roughness values found on the surface.

The effect of the casting technique on morphology is also crucial [66], as it can reveal information about the different phases. For example, Figure 4a,b shows a 3D representation of SEP copolymer (a) casted and (b) spin casted both from toluene and annealed at R.T. In Figure 4c,d, the 3D representation of the SI/sulf annealed at 80 °C and spin casted from (a) toluene and (b) THF can be observed to examine the differences in phase and topography.

The effect of the casting technique is obvious in Figure 4a,b, where lamellae morphology was observed for both samples, but phase differences are evident. For the casted sample, the roughness showed value of 0.094, while in the spin casted the roughness is increased to 0.157 due to the effect of the substrate. Interestingly, the skewness is less than 1, with values of 0.10 and 0.42, respectively, indicating almost symmetrical distribution of peaks and valleys, with the peaks being slightly more. Moreover, the thickness of the lamellas is also affected by the casting technique, showing 63 nm for the PS and 90 nm for the PEP for the casted samples, whereas for the spin-casted ones, the values are decreasing to 46 nm (PS) and 79 nm (PEP). These differences are due to the slower solvent evaporation rate from the casted technique that results in longer times for lamellas to develop and create thicker domains. For the spin-cast sample, it is also observed that the faster evaporation rate creates a more uneven distribution of the different phase values observed from the histogram in Figure 4b compared to Figure 4a.

On the other hand, from Figure 4c,d, the effect of the solvent evaporation of toluene (c) vs. THF (d) can be seen. The sample from toluene led to a not-fully organized structure, but with potential to become fully organized. In contrast, the THF one showed a lamellae morphology but was not fully organized. The not fully organized domains lead to smooth surfaces with roughness of 0.099 and 0.052, where the size of the domains can be found at 72 for the modified PI (Figure 4c) and 35 for the PS (Figure 4d). In addition, the sample spin-cast from THF has evaporated faster, leading to the formation of regions with potential crystallites, as indicated by the high percentage of one phase, which is likely due to the crystallite phase.

## 4. Conclusions

This work evaluated the morphologies of three copolymer samples by modifying only the PI segments (hydrogenation and sulfonation) of an initial PS-*b*-PI diblock copolymer. By adjusting casting techniques, annealing temperatures, and solvents, distinct topological structures were observed through AFM characterization. The different interactions between polymeric blocks and solvents over the various experimental parameter led to four primary morphologies: (i) lamellae, (ii) spherical micelles, (iii) worm-like, and (iv) network. Most samples displayed organized domains, primarily consisting of lamellae.

The spin-cast samples predominantly exhibited disordered morphologies, especially at 120 °C across all solvents. Cast samples showed alternating lamellae structures using toluene and THF at 80 °C. Cyclohexane produced spherical micelles (annealed at R.T. to 100 °C), while THF showed mainly worm-like structures. The chemical nature of the polymeric blocks also influences morphology, with the pristine diblock showing a lower interaction parameter than SEP, resulting in more disordered morphologies compared to the ordered structures of SEP. The SI/sulf copolymer’s interactions are complex due to multiple components—PI, PI/sulf, and PS—leading to intricate block/block and block/solvent interactions.

The sizes of organized domains depend on experimental parameters, with SEP micelles ranging from 73 nm to 80 nm and SI/sulf showing a similar trend. Using spin-casting keeps the PS domain size constant while increasing PEP up to 179 nm. The solvent also affected sizes.

The lamellae morphology of different copolymers revealed significant variations. PS domain sizes increased from 56 nm (SI) to 63 nm (SEP) and 82 nm (SI/sulf) respectively. In addition, PI’s initial size was 27 nm, changing to 90 nm (SEP) and 79 nm (SI/sulf).

Overall, it should be noted that the observed morphologies are specific to certain AFM experimental conditions, which is a limitation. Although the AFM is a powerful technique for characterizing the self-assembly of nanostructures, it has to be used in parallel with other techniques to verify these structures. A future perspective is the preparation of copolymers with three blocks: fully hydrogenated, partially sulfonated, and modified with hydrophobic segments.

## Figures and Tables

**Table 1:** Solubility parameter values for all solvents and polymeric blocks used.

Solvents Polymers	δ (MPa^1/2^) ^a^ (Hansen)	δ (MPa) ^b^ (Van Krelen)	δ (MPa^1/2^) ^b^ (Van Krelen)	δ (MPa) ^b^ (Van Krelen)
Solvents				
THF	19.46		19.50	
Toluene	18.16		18.20	
Cyclohexane	16.80		16.70	
Polymer blocks				
PS	19.07		19.10	
PI	17.94		17.40	
PEP	17.47 ^c^		16.86 ^d^	
PI/sulf	24.88 ^c^		26.44 ^d^	

^a^ Overall solubility was calculated from δ^2^ = δ_d_^2^ + δ_p_^2^ + δ_h_^2^ [51]. ^b^ Solubility values were found in the literature or calculated from Van Krelen [53]. ^c^ Solubility values were calculated from contributions of sulfonated PI groups by δ_d_ = ΣF_di_/V, δ_p_ = √ΣF_pi_^2^/V and δ_h_ = √ΣE_di_/√V [53]. The rest were found in the literature [51]. Volume values were also calculated from the contributions of each group of the chemical structure [53]. ^d^ Solubility values were calculated from δ = (E_coh_/V)^1/2^ by using contributions of each group to E_coh_ [53].

**Table 2:** χ_AΒ_ and χ_SP_ values for all solvents and blocks used for all samples.

χ_AB_ ^a^	298 K (R.T.)	χ_SP_ ^b^	THF	Toluene	Cyclohexane
χ_PS/PI_	0.05	χ_PS_	0.09	0.17	0.36
χ_PS/PEP_	0.10	χ_PI_	0.16	0.04	0.13
χ_PS/PI/sulf_	1.35	χ_PEP_	0.20	0.12	0.16
χ_PI/PI/sulf_	1.93	χ_PI/sulf_	0.34	1.26	1.80

^a^ χ_AB_ values were calculated from χ_AB_ ≈ (δ_A_ − δ_B_)^2^V_reff_/RT equation, where V_reff_ = 100 cm^3^/mol, R = 8.314 cm^3^ MPa/K·mol and δ in MPa taken from Table 1 (Hansen). ^b^ χ_SP_ values were calculated from χ_sp_ = (aV/RT)[(δ_ds_ − δ_dp_)^2^ + 0.25(δ_ps_ − δ_pp_)^2^ + 0.25(δ_hs_ − δ_hp_)^2^] equation, where V is in cm^3^/mol, R = 8.314 cm^3^ MPa/K·mol, a is constant equal to 0.6 [54], and δ_d_, δ_p_, and δ_h_ in MPa^1/2^ were taken also from the literature [53].

**Table 3:** Characteristic morphologies observed for all samples at different conditions.

	Copolymers					
	SI		SEP		SI/sulf	
	Casting Technique					
	Casting	Spin Casting	Casting	Spin Casting	Casting	Spin Casting
Morphologies	Samples		Samples		Samples	
Disorder	* 2C, 2F, 3C 3T, 3F, 4C 4T, 4F	2F, 3C, 3T 4C, 4T, 4F	3T	2C, 4T, 4F	2C, 2T, 4C 4T, 4F	1F, 4C, 4T 4F
Lamellae	-	3F	1T, 2T, 2F 4T	1T	2F	-
Lamellae (not fully organized)	1T, 1F, 2T	1T	1F	-	1T, 1F	-
Lamellae (not fully organized) with crystallites	-	2T	-	3T	-	1T, 2T, 2F
Network	-	-	4C, 4F	4C	-	3T
Spherical micelles	-	1C, 2C	1C, 2C, 3C	1C	3C 3T, 3F	-
Spherical micelles with crystallites	-	-	-	-	-	1C, 2C, 3F
Worm-like	1C	1F	3F	1F, 2T, 2F 3C	1C	3C
Worm-like with crystallites	-	-	-	3F	-	-

* Table is divided for different samples as long as for casting (c) and spin casting (sc) techniques. Abbreviations for each sample are referred as XY, where X is from 1–4 (R.T., 80, 100, 120 °C) and Y is for different solvents: C (cyclohexane), T (toluene) and F (THF).

## Data Availability

The original contributions presented in this study are included in the article/Supplementary Materials. Further inquiries can be directed to the corresponding author.

## Supplementary Materials

The following supporting information can be downloaded at: https://www.mdpi.com/article/10.3390/polym17223047/s1, Figure S1: ^1^H-NMR for the sulfonation of the sample SI; Figure S2: All morphologies observed by AFM phase images (3 μm × 3 μm) with a 3D representation of a 1 μm × 1 μm and a profile of the morphology of 1.5 μm diagonal; Table S1: Characteristic values of roughness Sa, skewness Sk and domain sizes of bright and dark areas for all samples. The abbreviation for the samples is sample-solvent-casting technique-annealing temperature.
