# Supplementary material for: Morphological Characterization of High Molecular Weight Poly(styrene-b-isoprene) or PS-b-PI and Its Hydrogenated, Sulfonated Derivatives: An AFM Study
Source: Polymers (Basel). 2025 Nov 17;17(22):3047. doi: 10.3390/polym17223047 (PMC12656379; doi:10.3390/polym17223047)
Supplement: Supplementary file 1 [file polymers-17-03047-s001.zip › polymers-3951950-supplementary.pdf]

Morphological characterization of high molecular weight poly(styrene-b-isoprene) (PS-b-PI), and its hydrogenated, sulfonated derivatives: an AFM study

Materials and Methods

The molecular characterization of the initial copolymer and its derivatives was conducted by size exclusion chromatography using a PerkinElmer (Waltham, MA, USA) chromatograph equipped with a binary pump and a refractive index (RI) detector. The eluent used was THF and separation was carried out with four columns packed with particle gels bearing different nominal pore sizes. The elution rate was 1 mL/min at 30 °C. The molecular weights were calculated based on a calibration curve from monodisperse polystyrene standards.

Infrared spectroscopy (FT-IR) was performed with a Nicolet Nexus 670 (Wake Forest, NC, USA) spectrometer equipped with a single horizontal golden gate attenuated total reflectance (ATR) cell. Spectra were recorded by averaging 20 scans between 4000 and 400 cm−1 with a resolution of 2 cm−1 under ambient conditions.

Results and Discussion

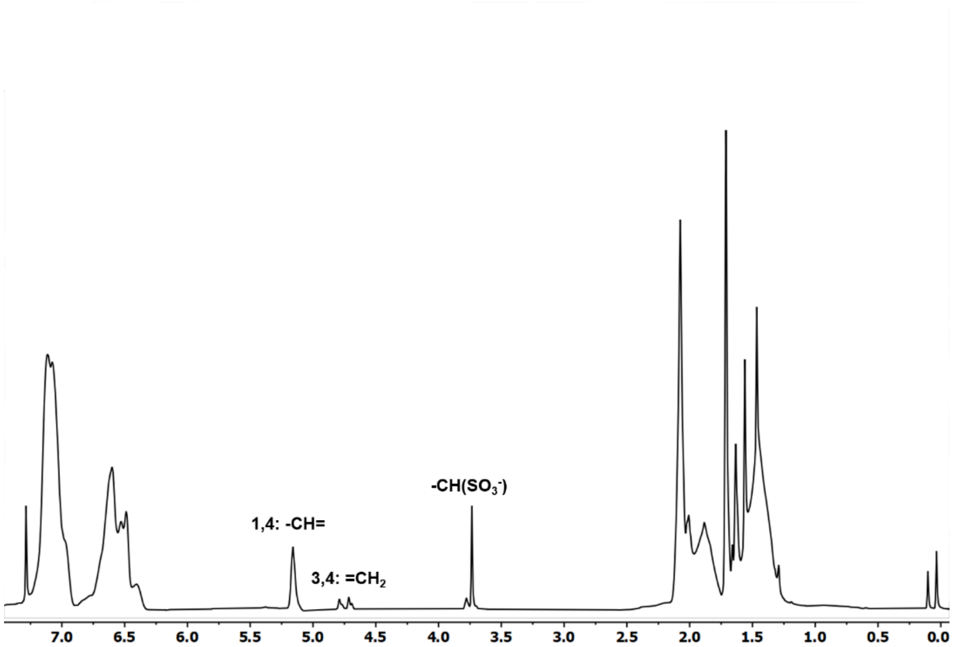

Figure S1. ^{1}H-NMR for the sulfonation of the sample SI.

From the ^{1}H-NMR of the sulfonated block copolymer the most important is the formation of new chemical shifts since the alteration of the chemical structure and the presence of the sulfonyl groups in the PI chain. These new shifts can be found on 3.75 ppm (-CH(SO_3^-)-), indicating the success of the sulfonation and also on 2.00 ppm where the -OH group attached to the PI chain can be found. Since the sulfonation was not 100% (as was expected), characteristic peaks for the PI at ~5.00 ppm observed. By integrating and incorporate the molecular weights for the monomeric units for both structures for the region 5.10 ppm (for the PI) and the 3.75 ppm (for the sulfonated PI) the sulfonation degree can be found at ~43%.

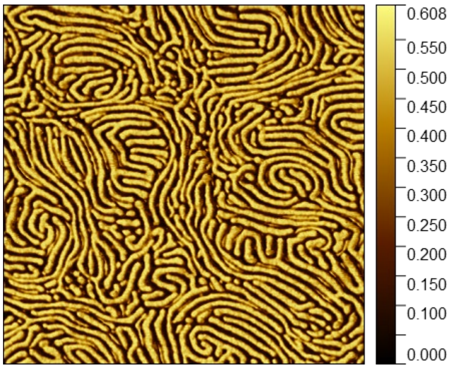

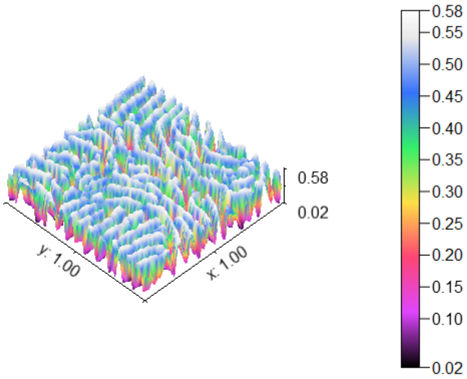

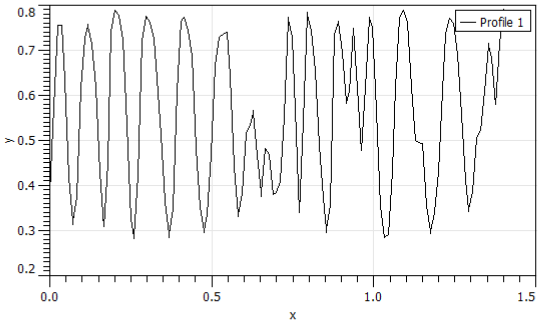

SI-cyclohexane-casted-RT

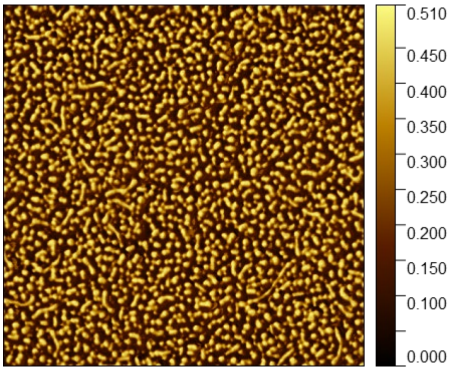

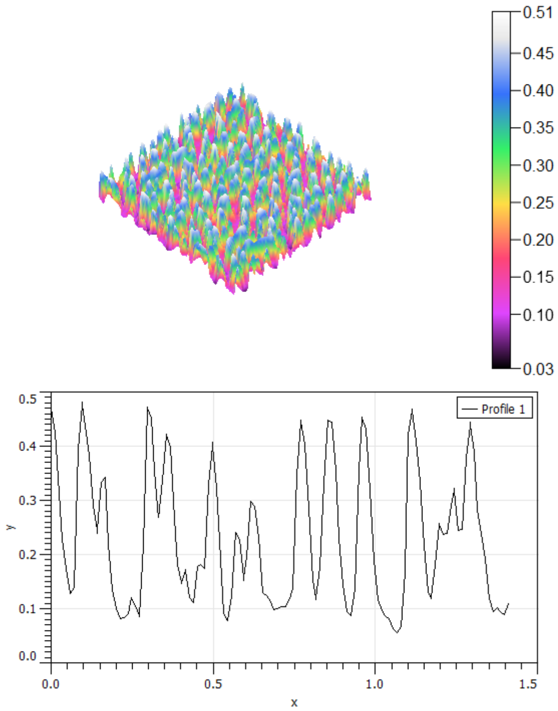

SI-cyclohexane-spin casted-RT

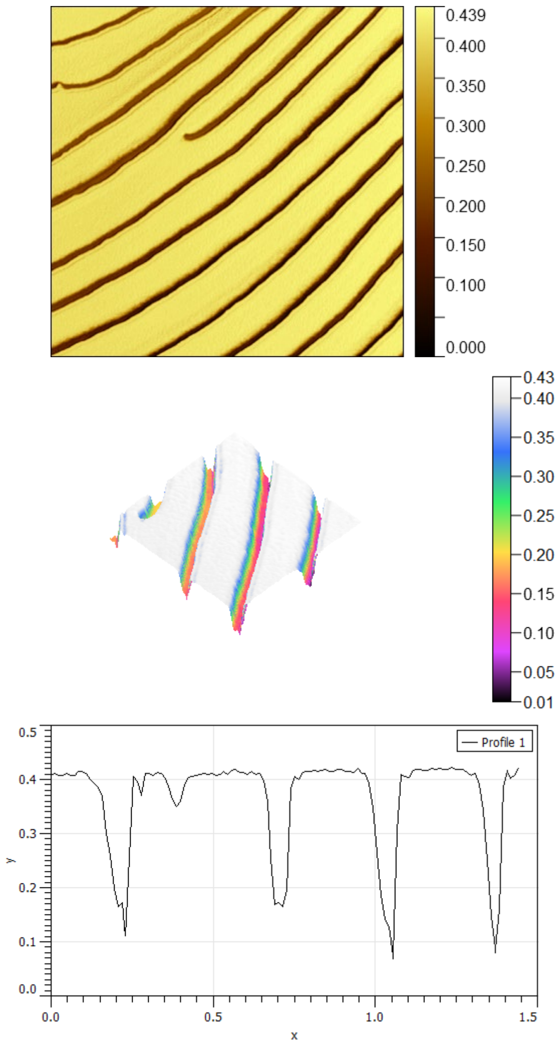

SI-toluene-casted-RT

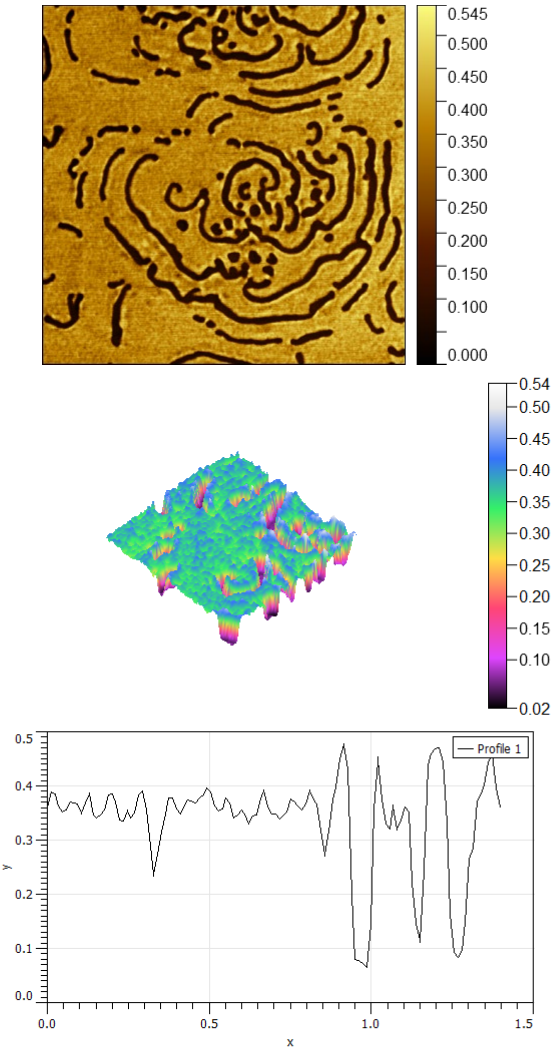

SI-toluene-spin casted-RT

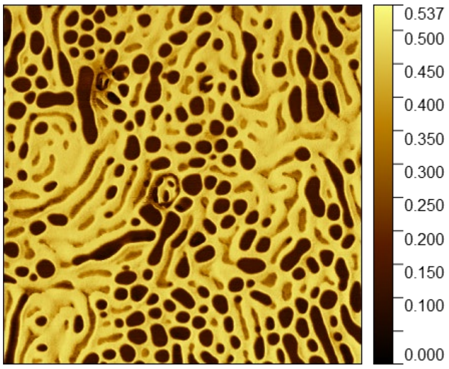

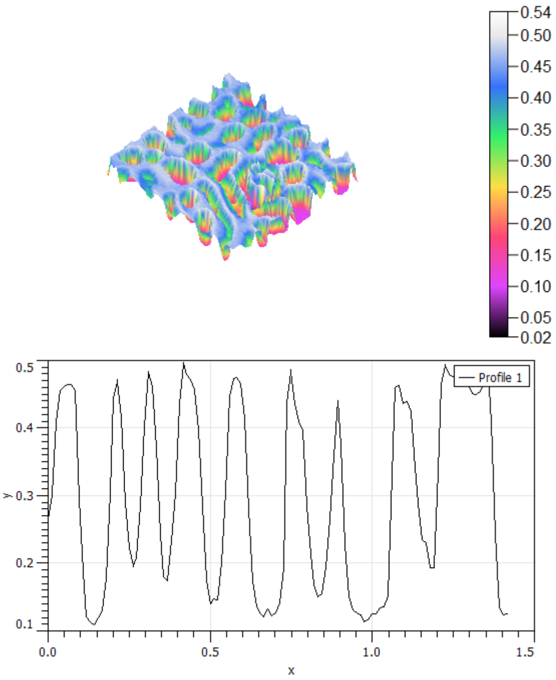

SI-thf-casted-RT

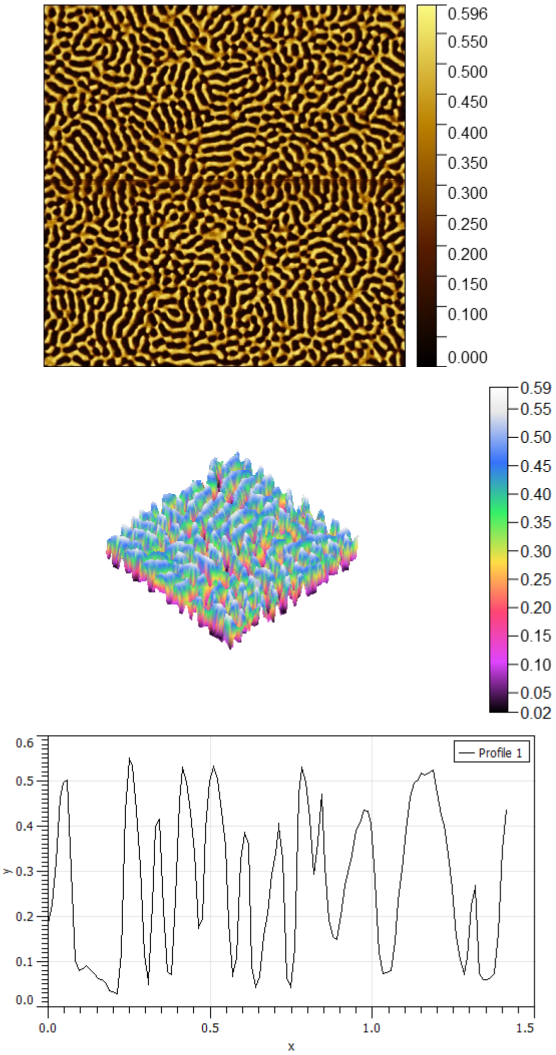

SI-thf-spin casted-RT

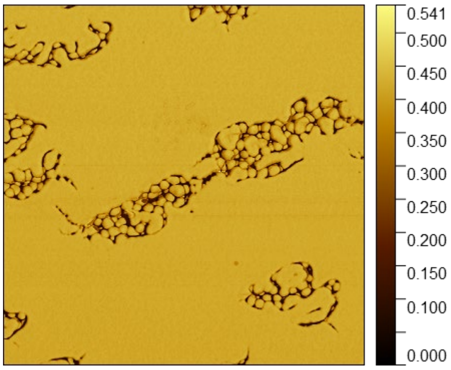

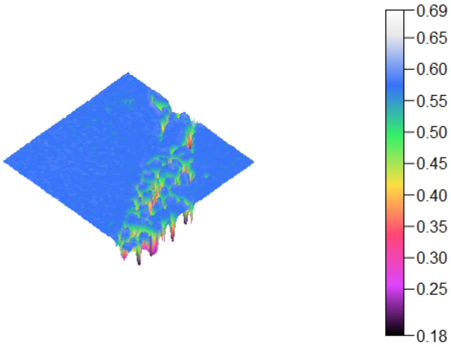

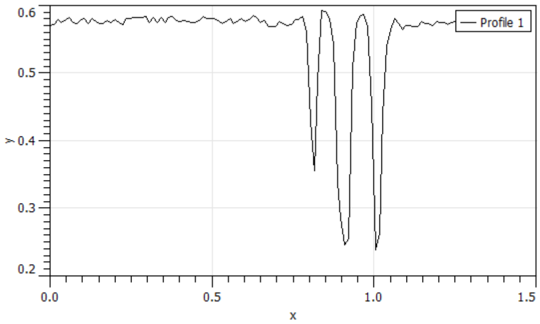

SI-cyclohexane-casted-80

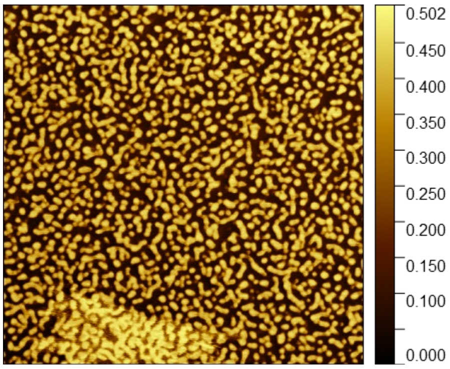

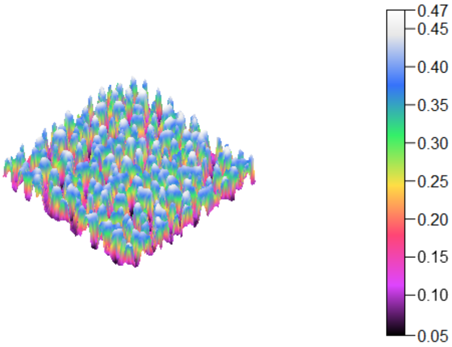

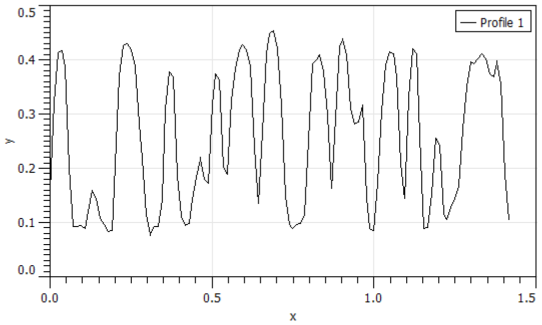

SI-cyclohexane-spin casted-80

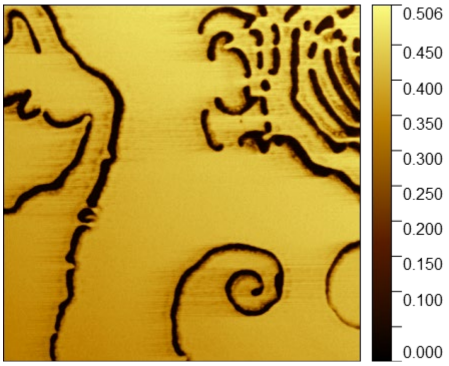

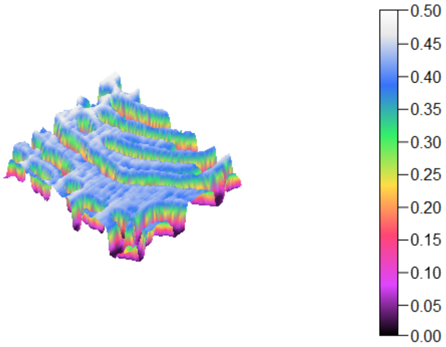

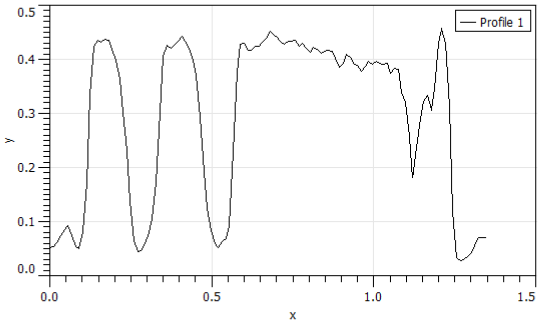

SI-toluene-casted-80

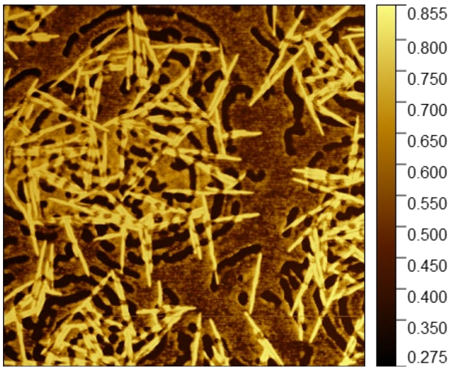

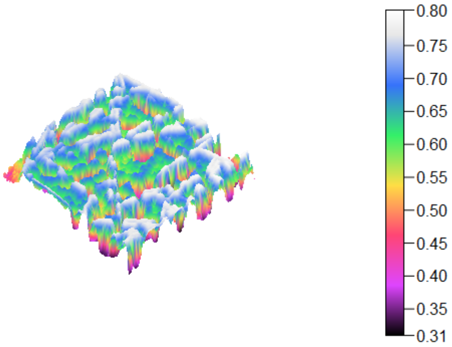

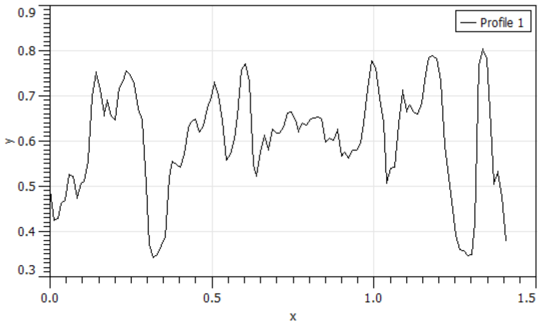

SI-toluene-spin casted-80

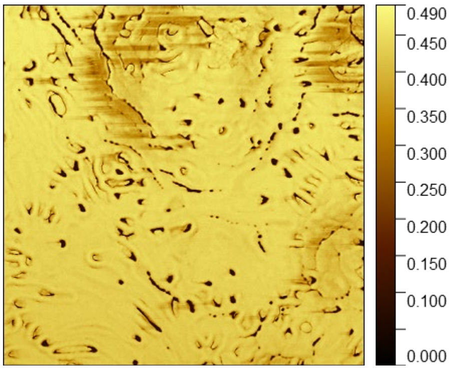

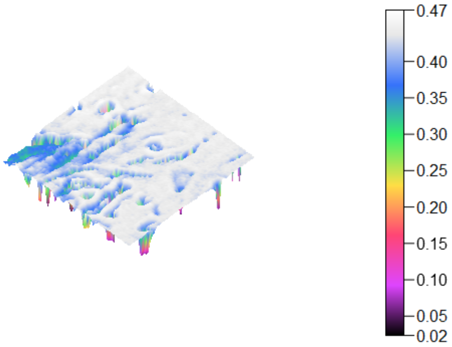

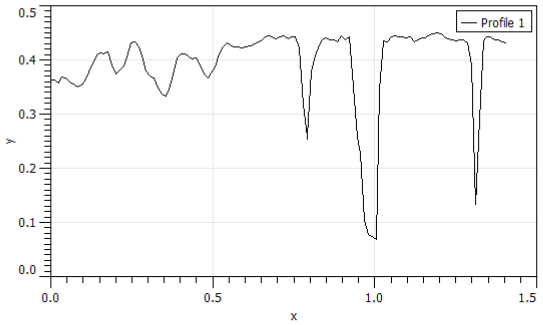

SI-thf-casted-80

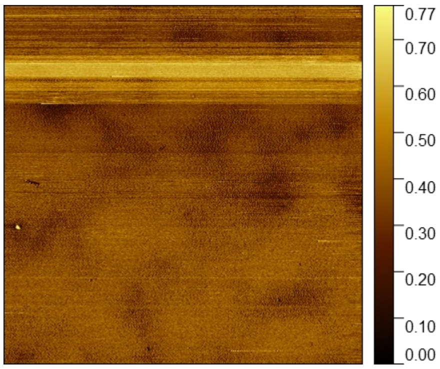

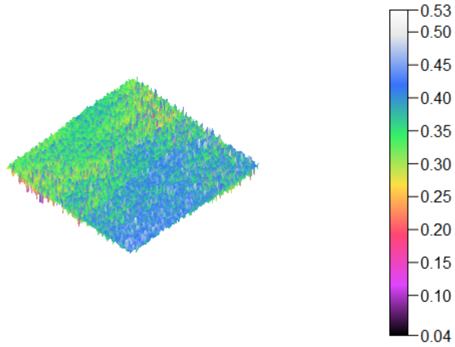

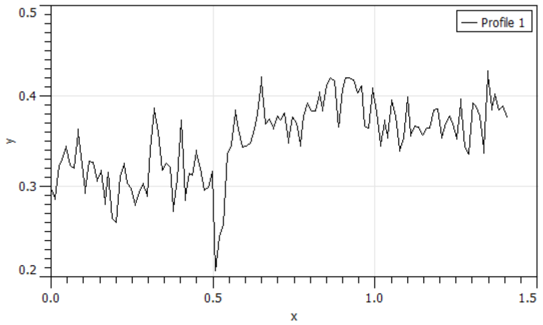

SI-thf-spin casted-80

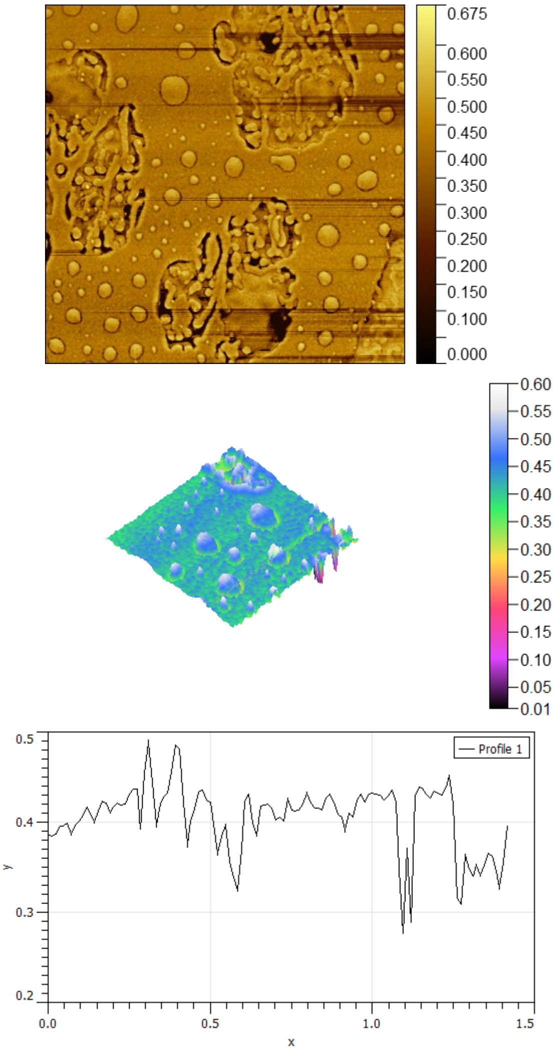

SI-cyclohexane-casted-100

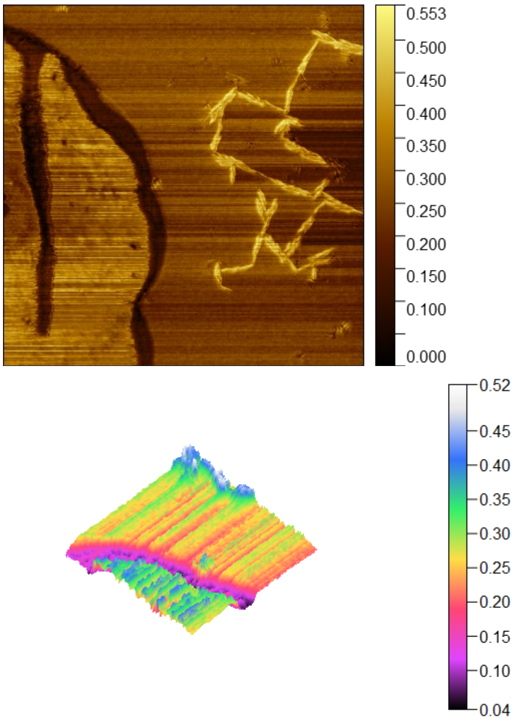

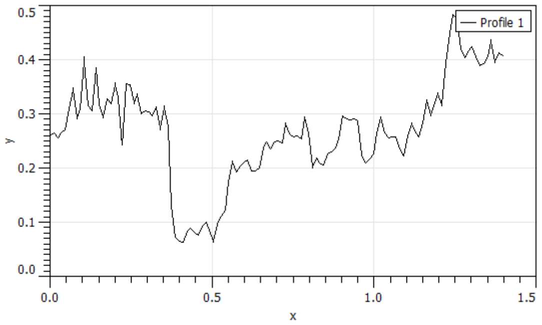

SI-cyclohexane-spin casted-100

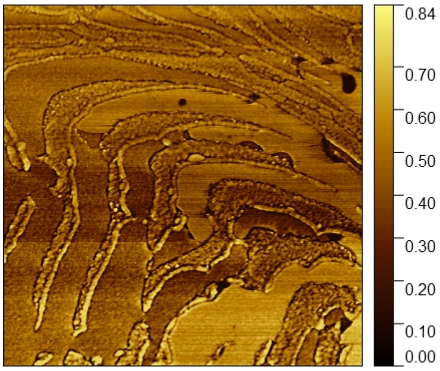

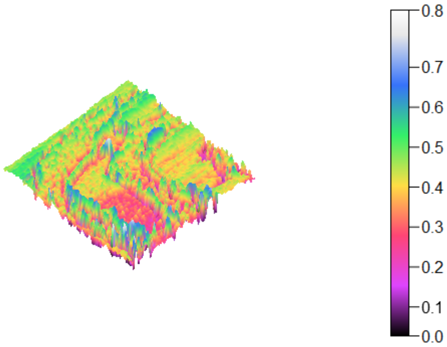

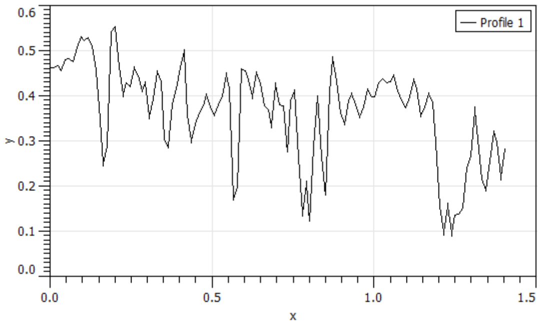

SI-toluene-casted-100

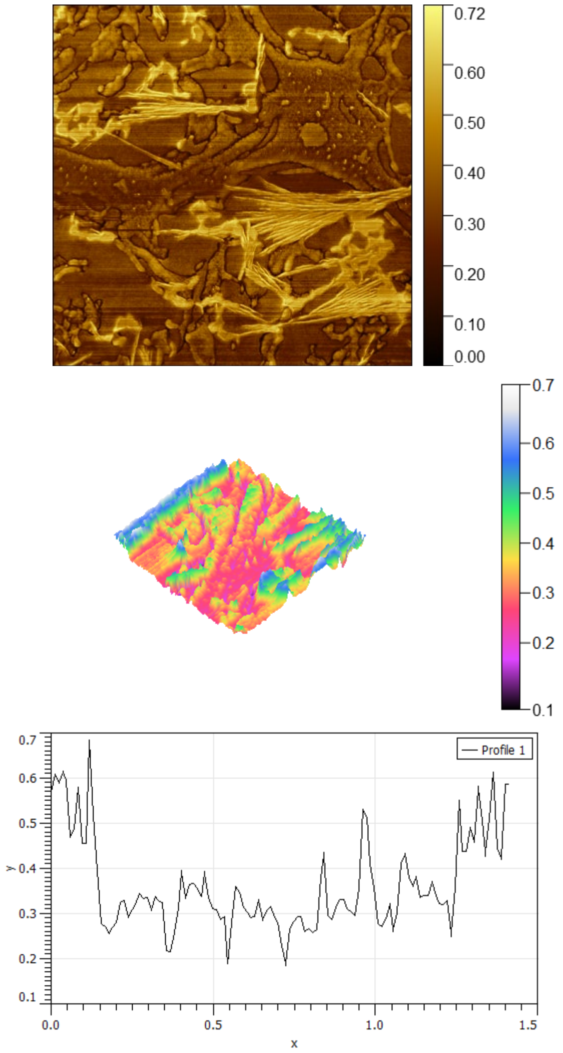

SI-toluene-spin casted-100

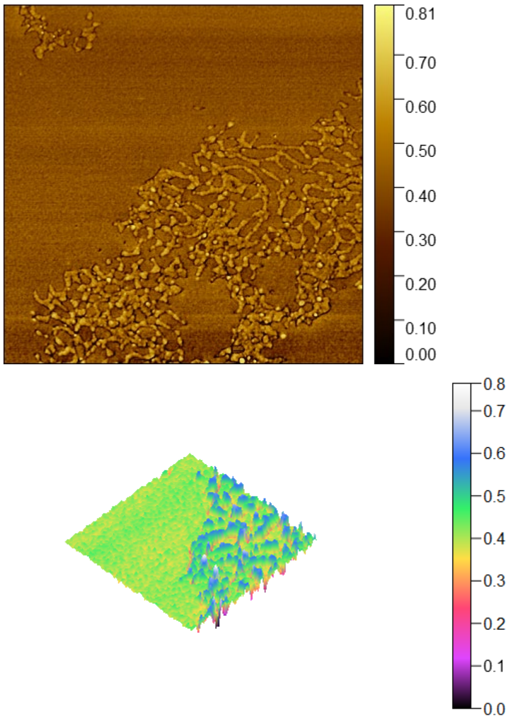

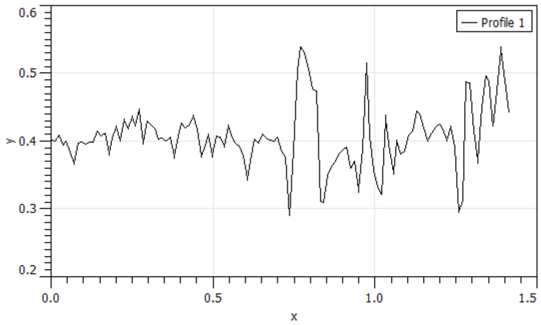

SI-thf-casted-100

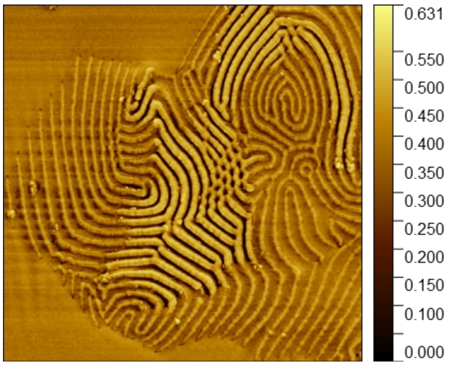

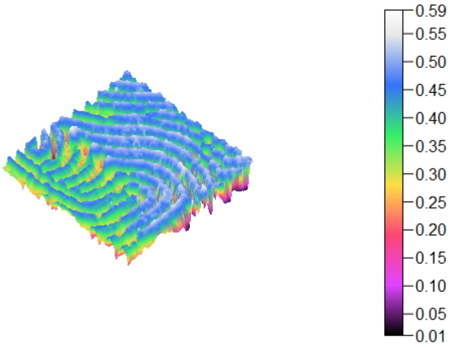

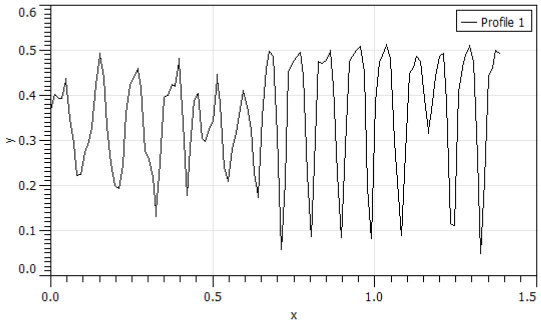

SI-thf-spin casted-100

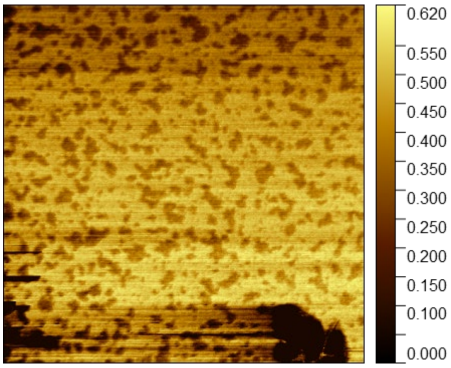

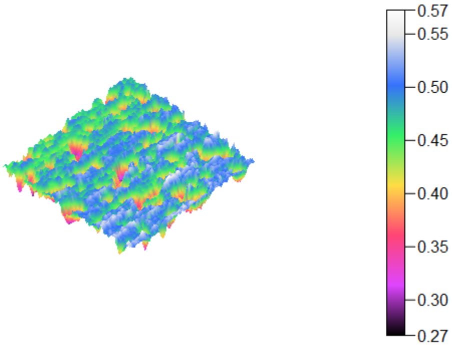

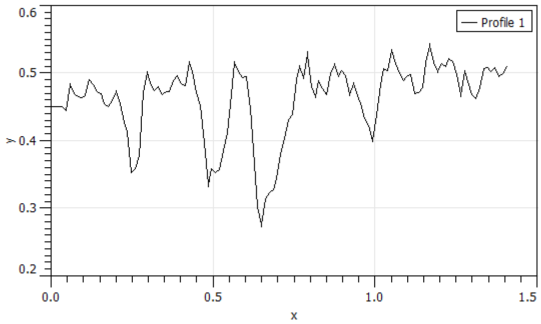

SI-cyclohexane-casted-120

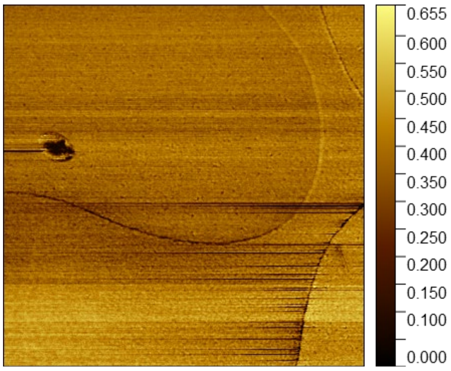

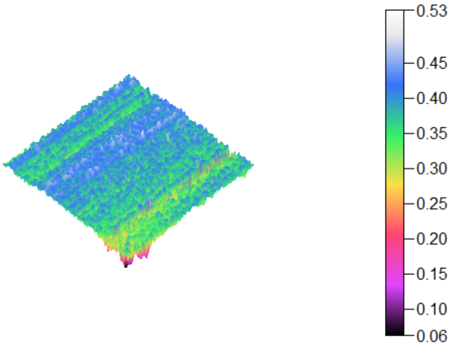

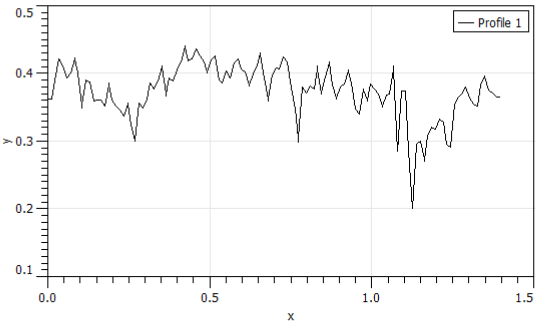

SI-cyclohexane-spin casted-120

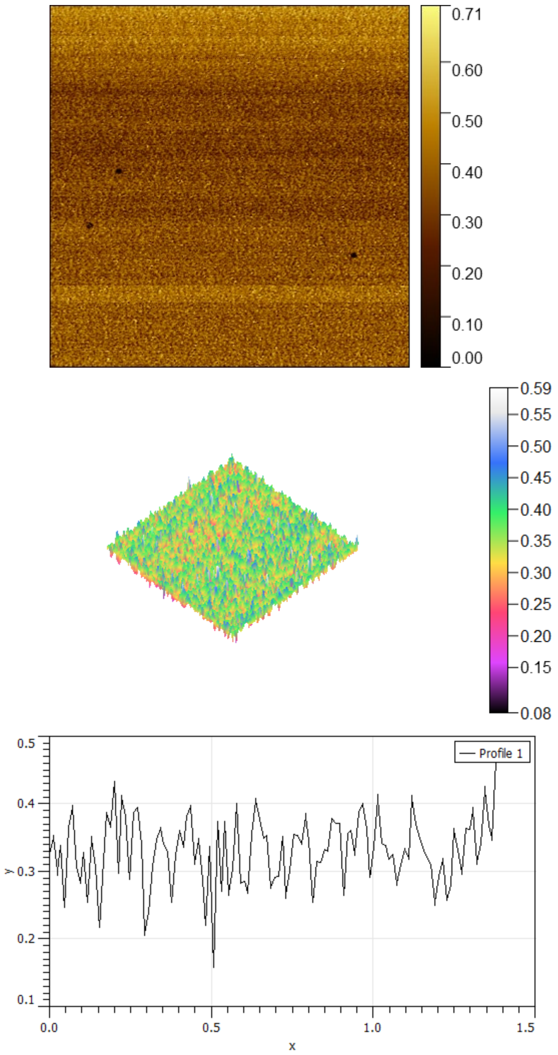

SI-toluene-casted-120

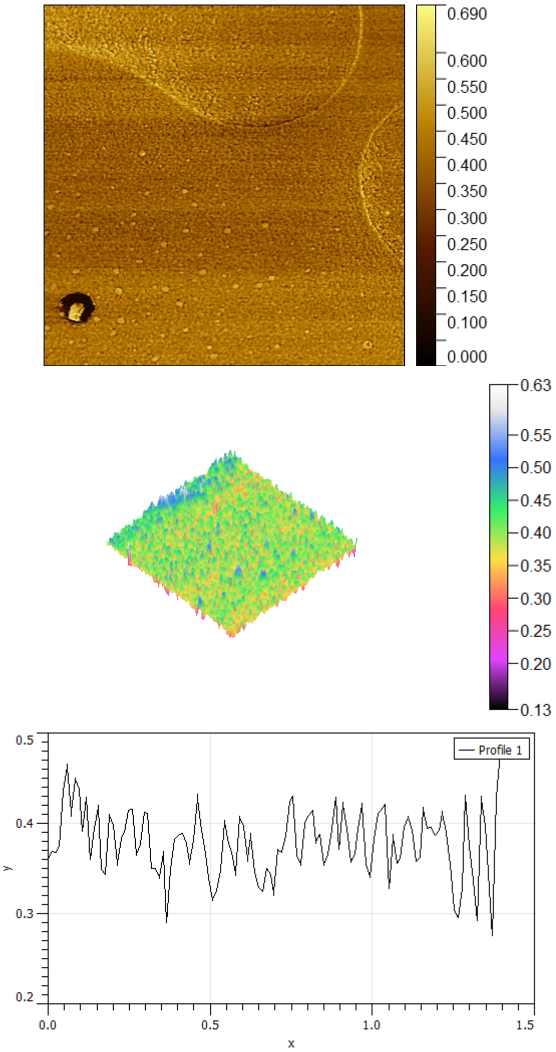

SI-toluene-spin casted-120

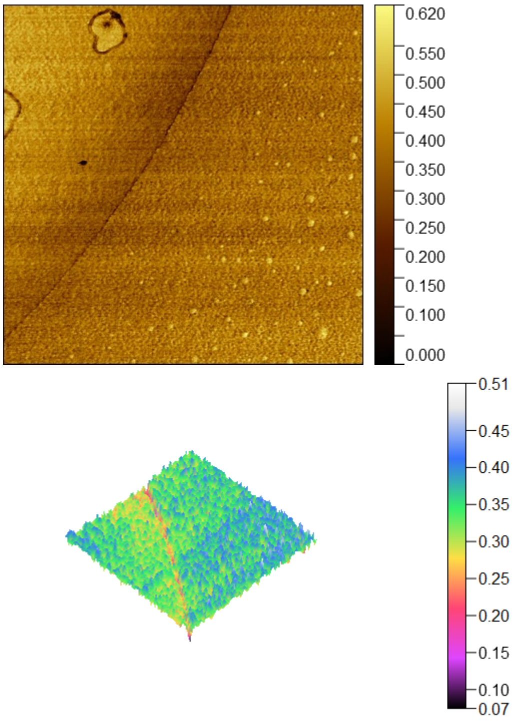

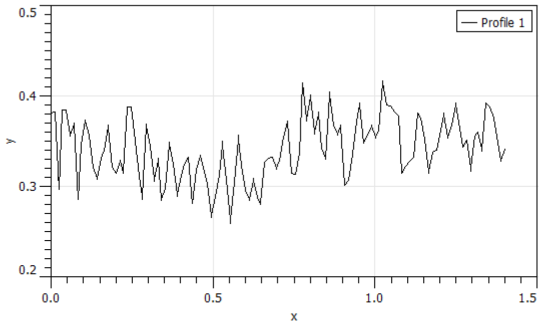

SI-thf-casted-120

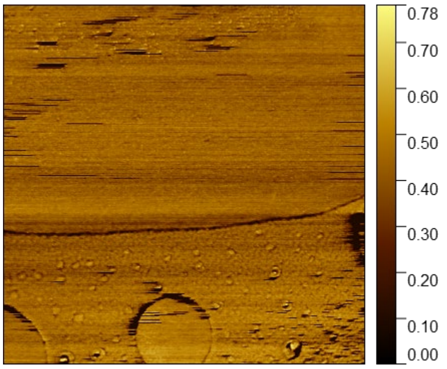

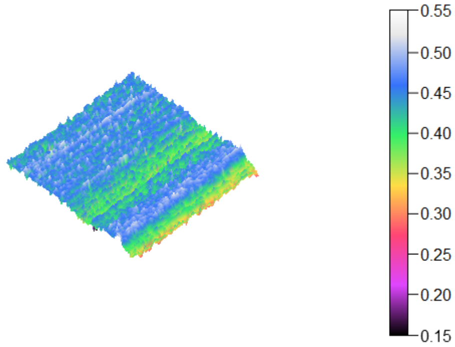

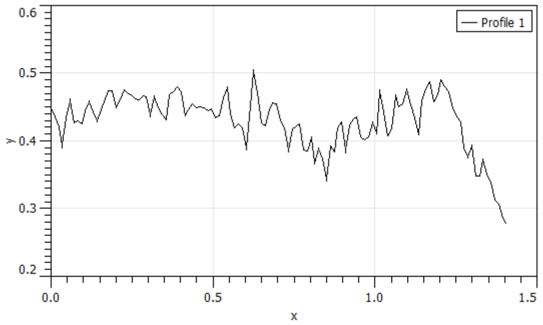

SI-thf-spin casted-120

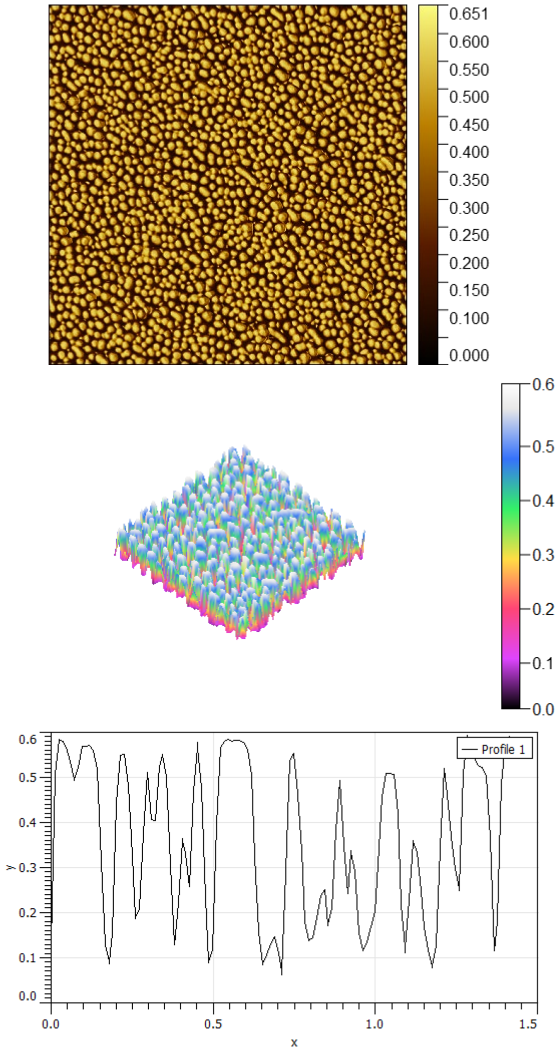

SEP-cyclohexane-casted-RT

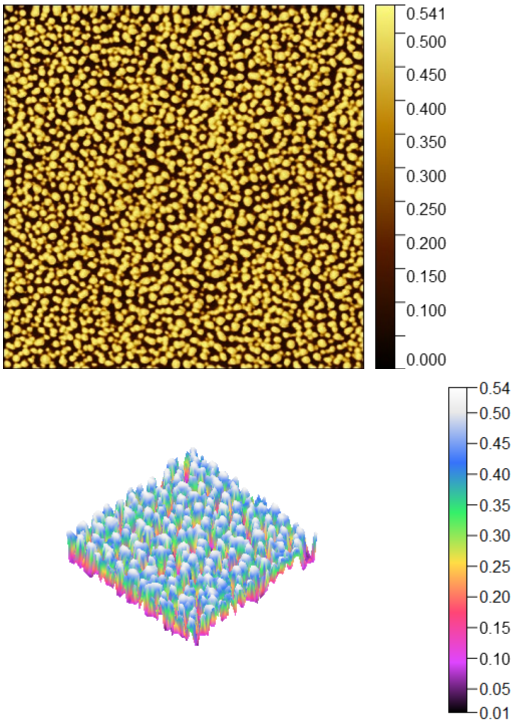

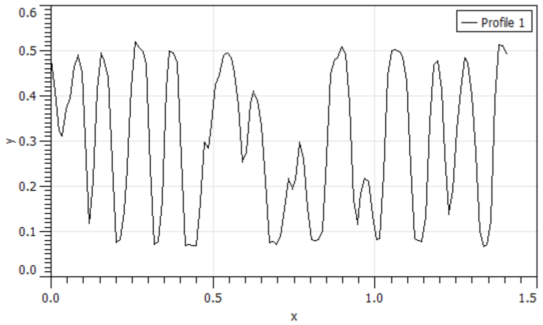

SEP-cyclohexane-spin casted-RT

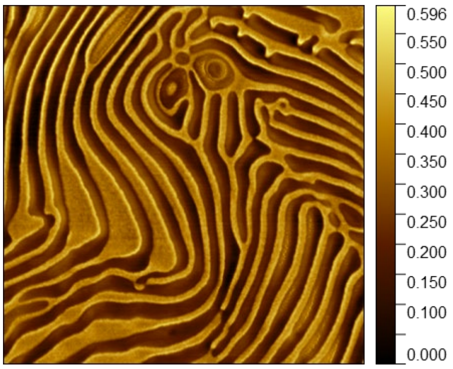

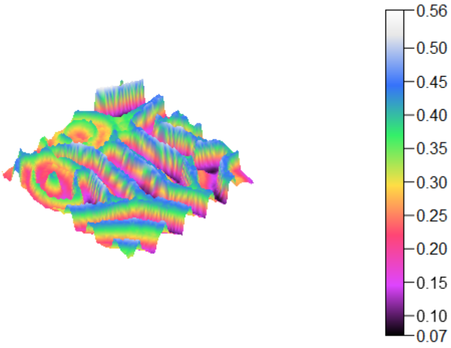

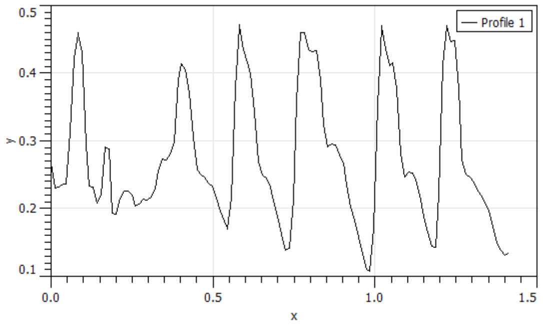

SEP-toluene-casted-RT

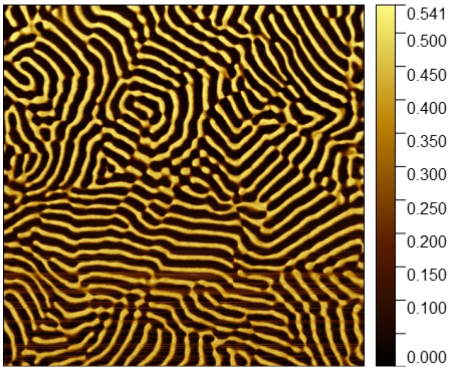

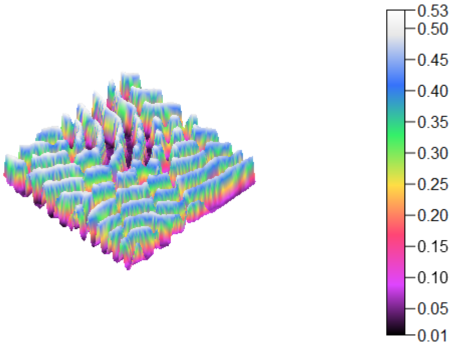

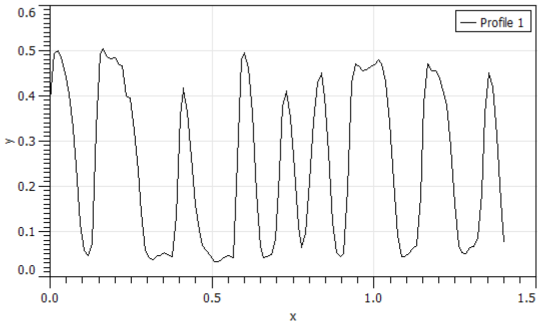

SEP-toluene-spin casted-RT

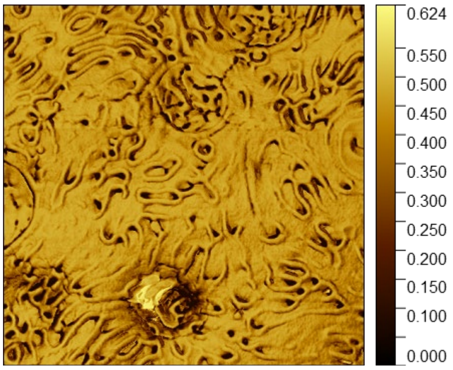

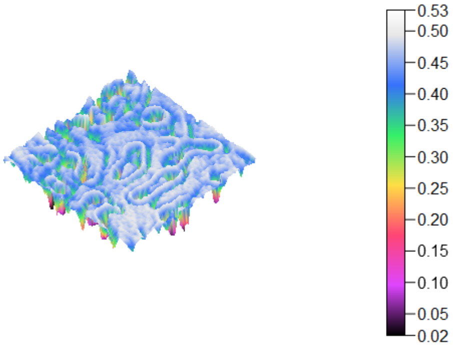

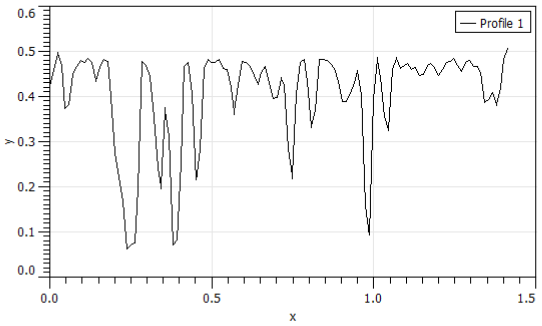

SEP-thf-casted-RT

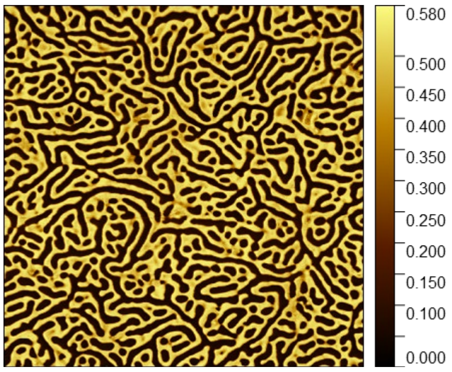

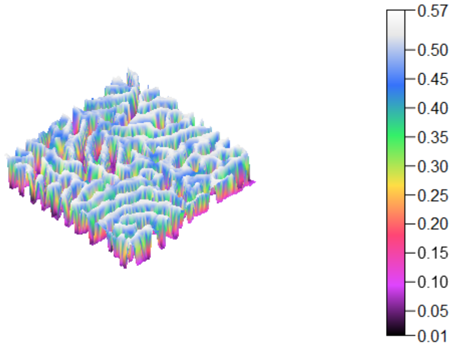

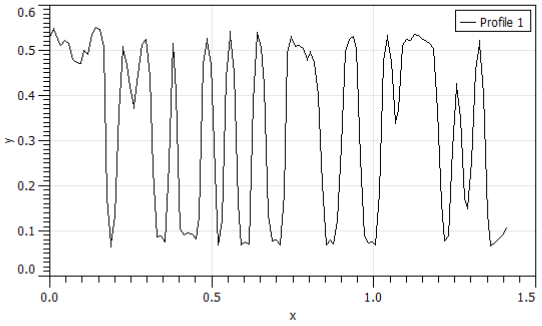

SEP-thf-spin casted-RT

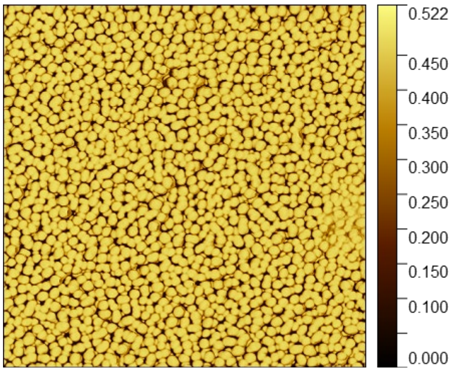

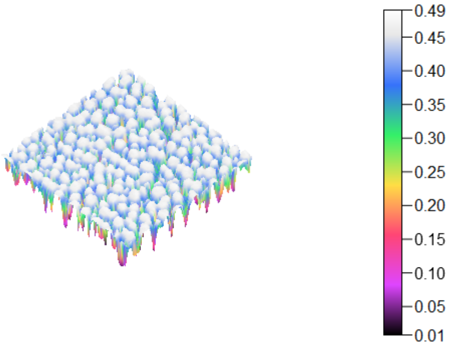

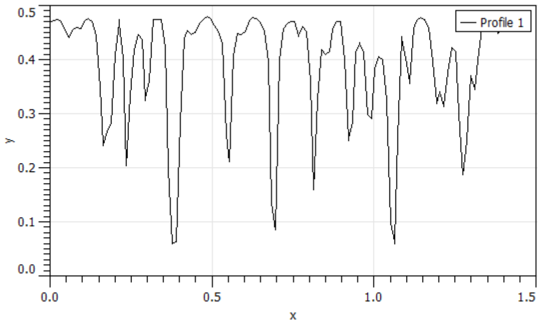

SEP-cyclohexane-casted-80

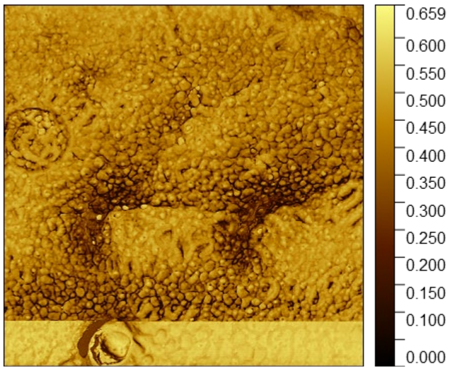

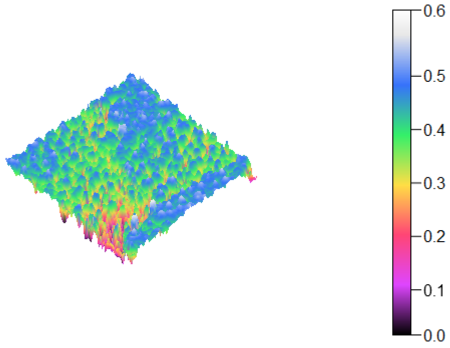

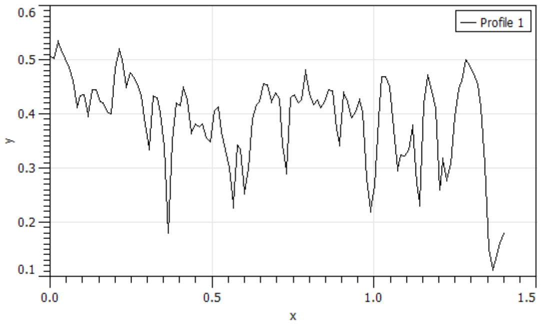

SEP-cyclohexane-spin casted-80

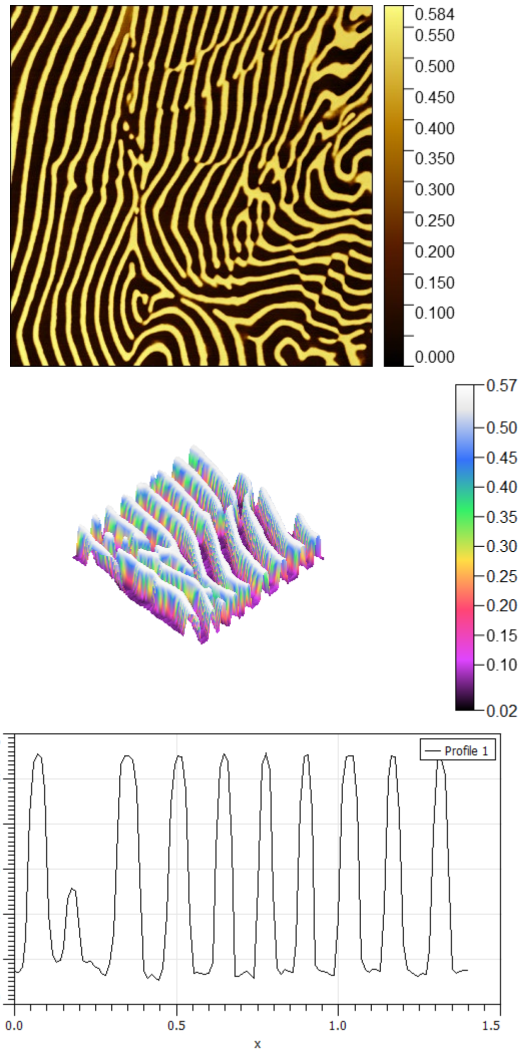

SEP-toluene-casted-80

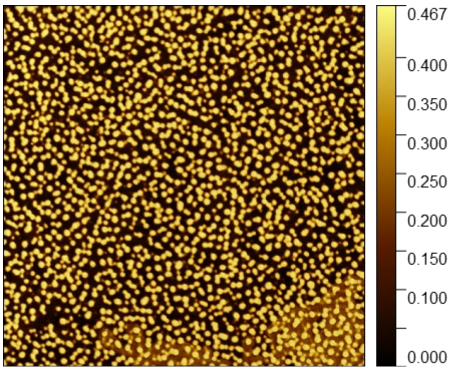

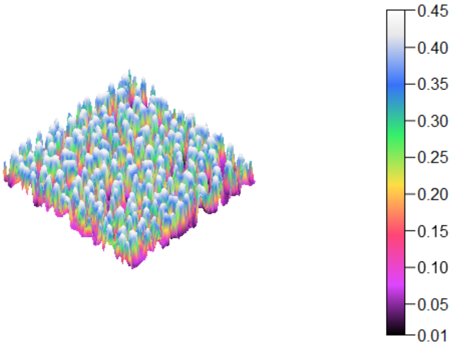

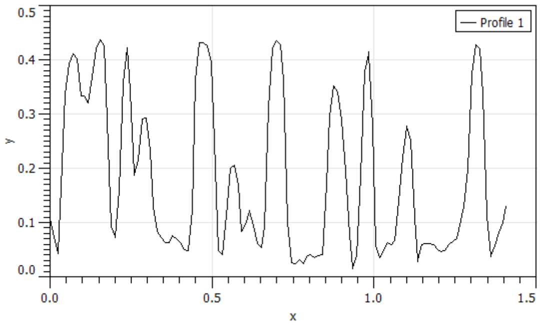

SEP-toluene-spin casted-80

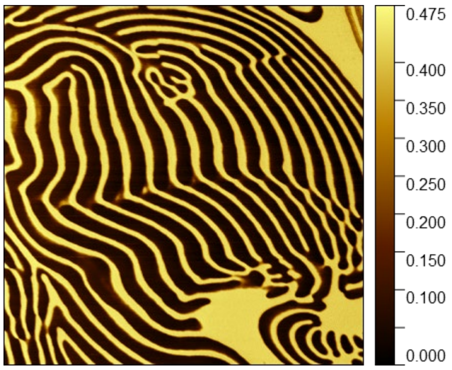

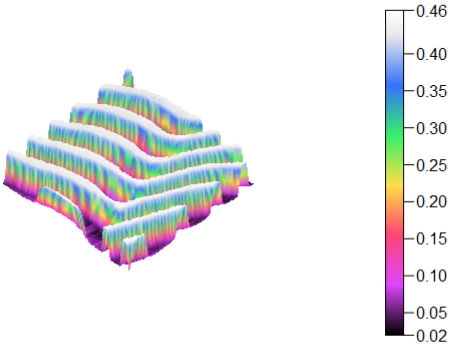

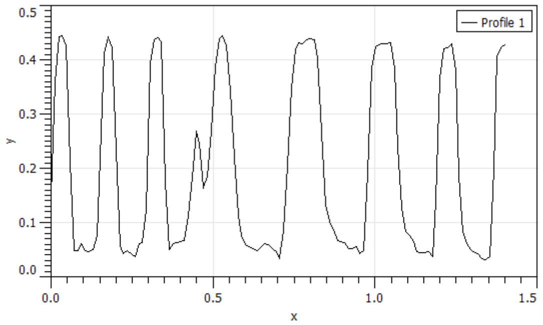

SEP-thf-casted-80

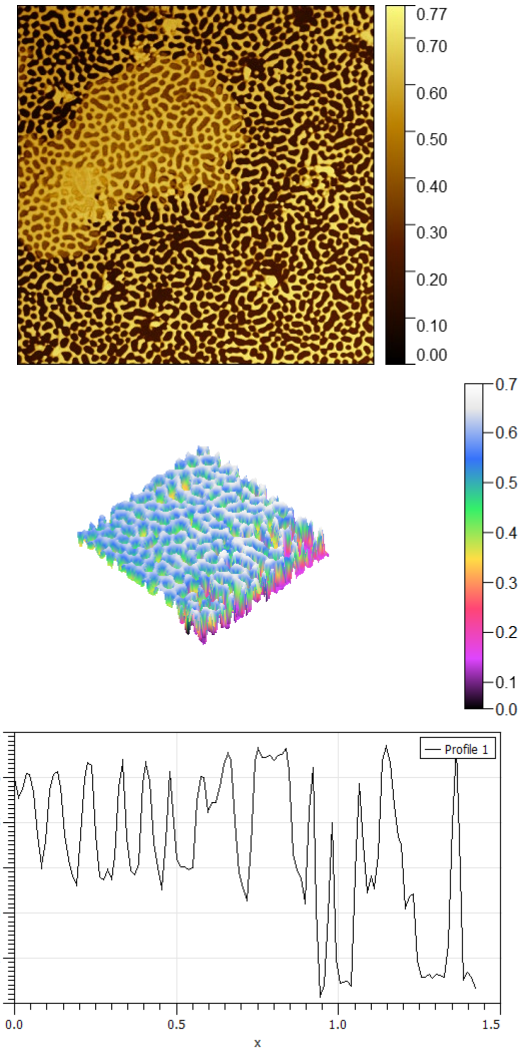

SEP-thf-spin casted-80

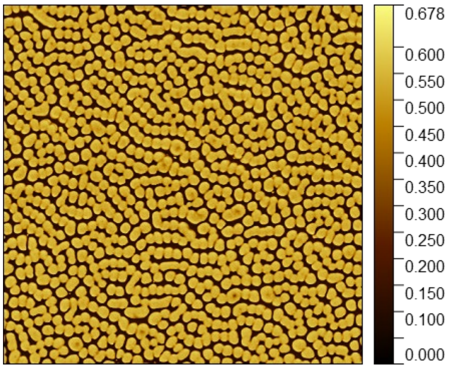

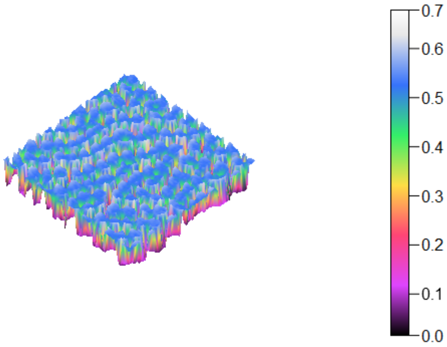

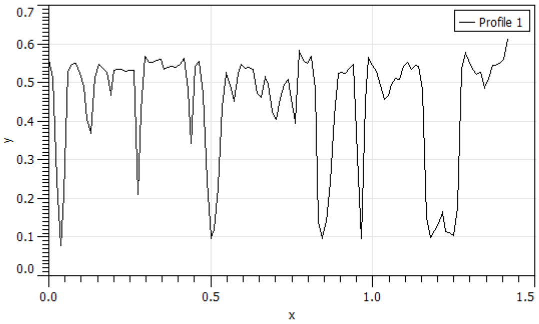

SEP-cyclohexane-casted-100

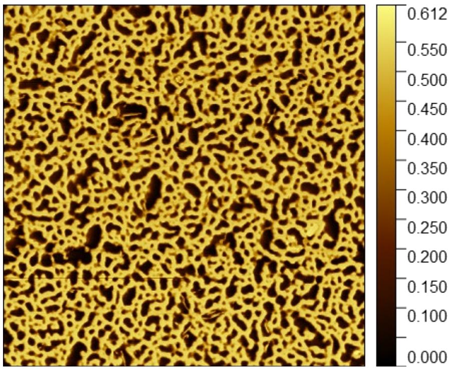

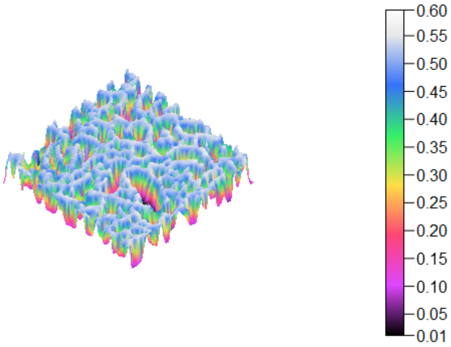

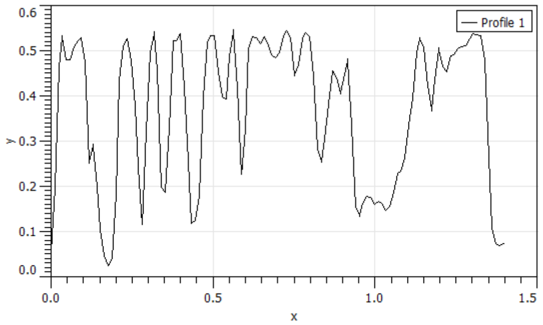

SEP-cyclohexane-spin casted-100

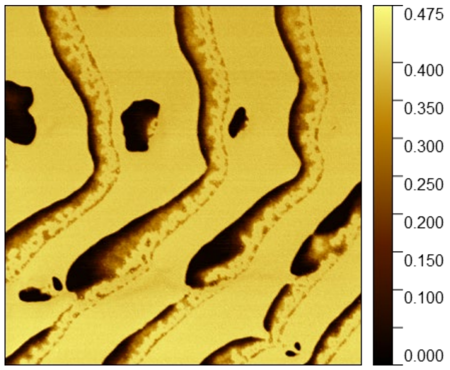

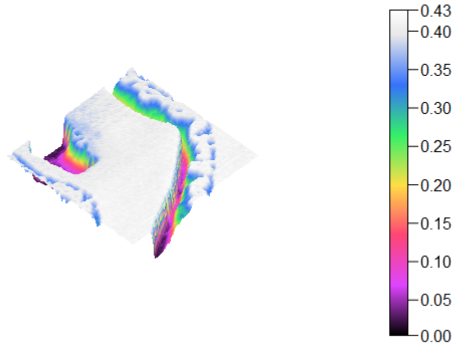

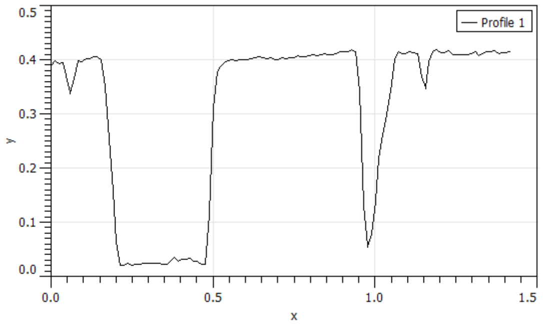

SEP-toluene-casted-100

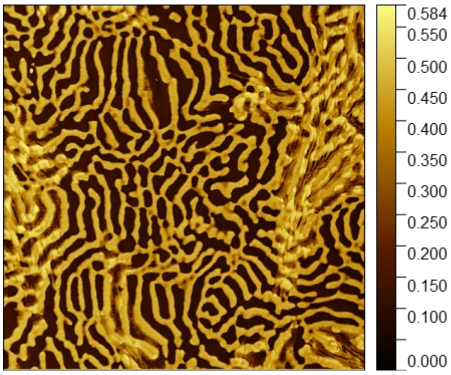

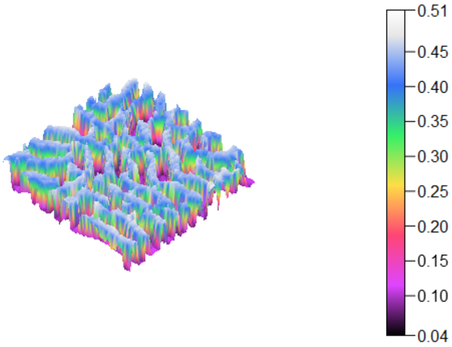

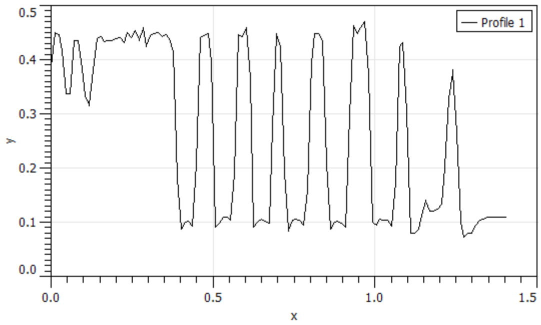

SEP-toluene-spin casted-100

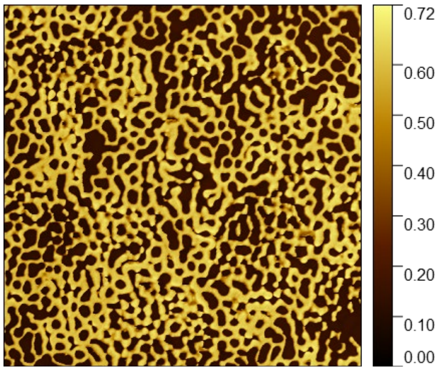

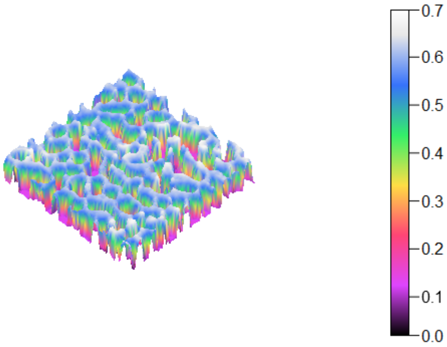

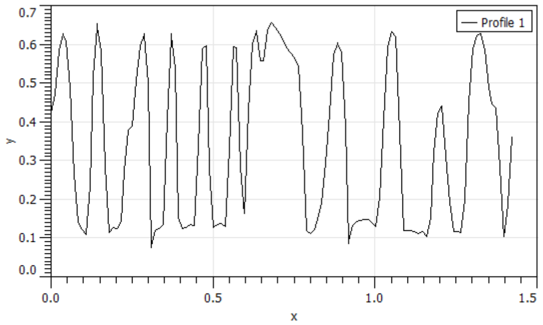

SEP-thf-casted-100

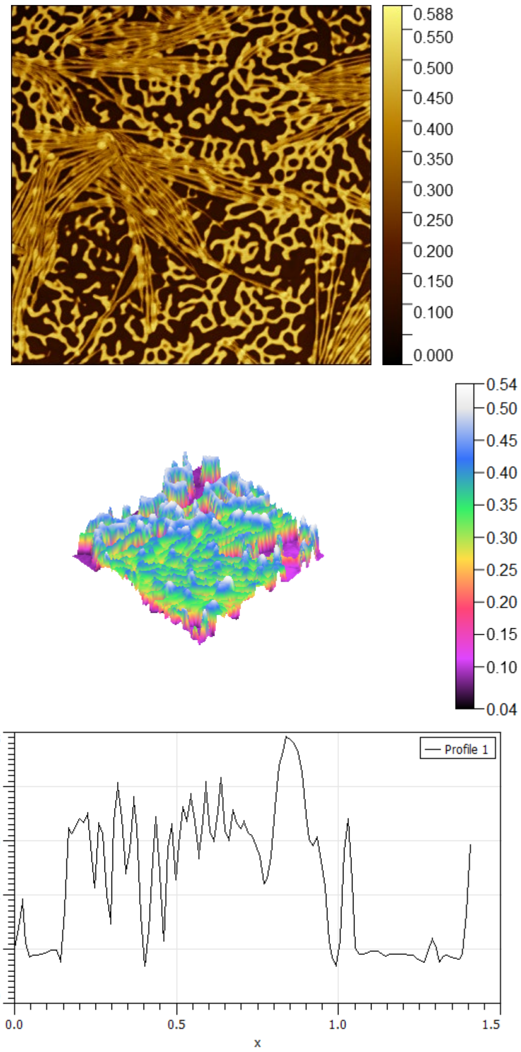

SEP-thf-spin casted-100

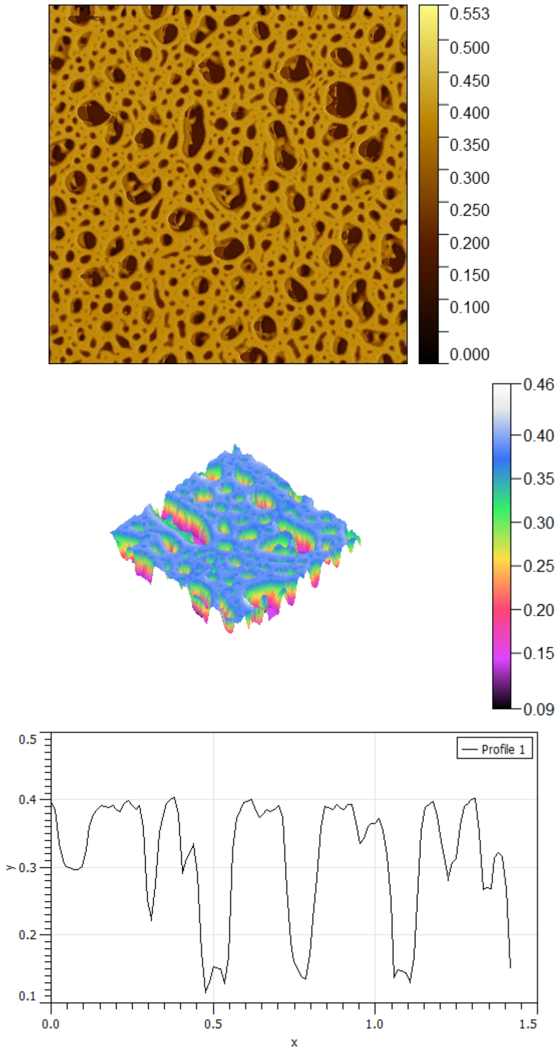

SEP-cyclohexane-casted-120

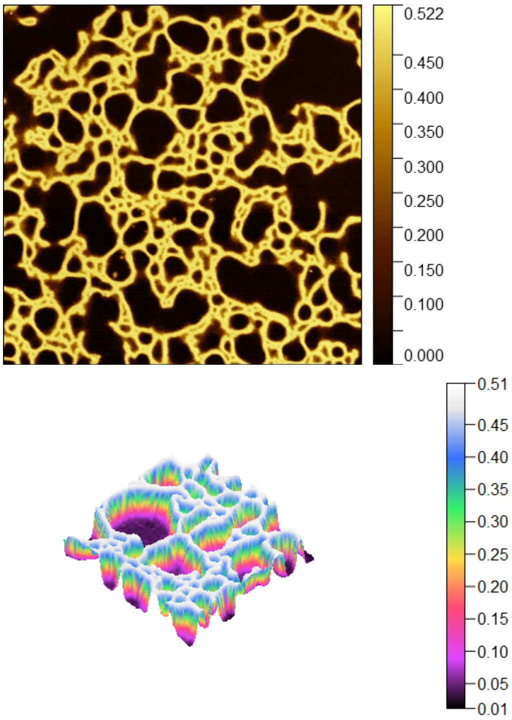

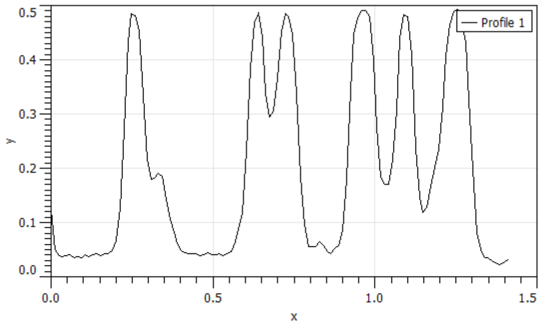

SEP-cyclohexane-spin casted-120

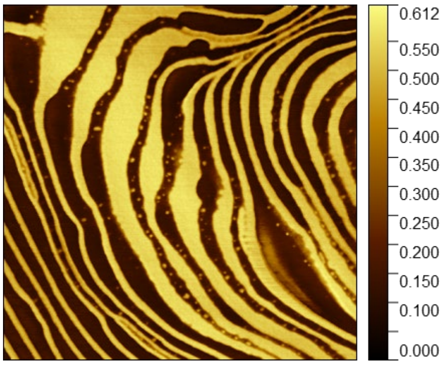

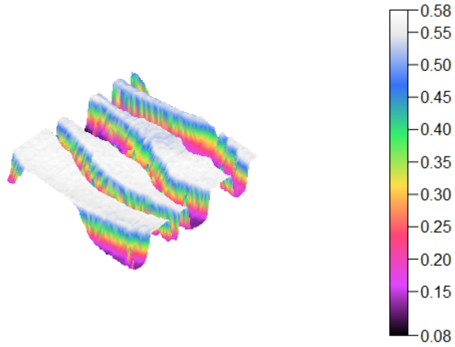

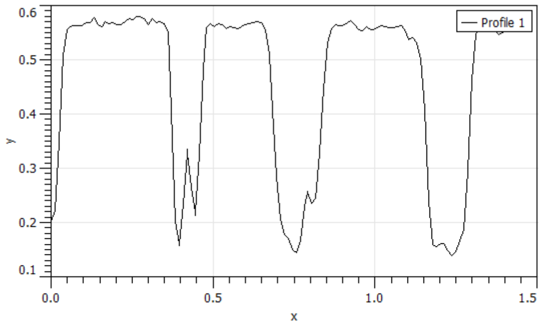

SEP-toluene-casted-120

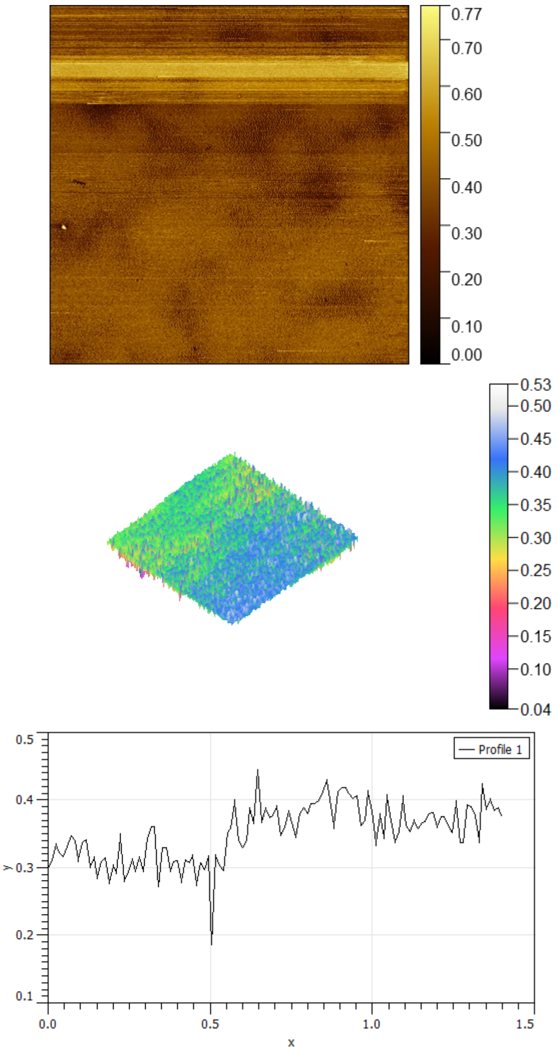

SEP-toluene-spin casted-120

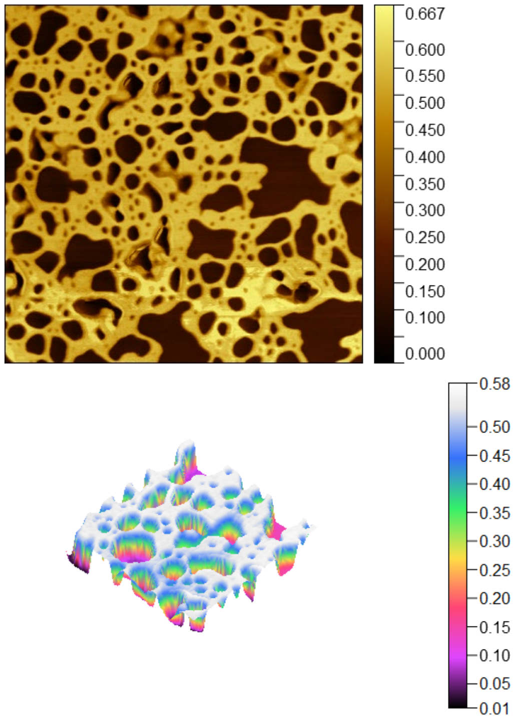

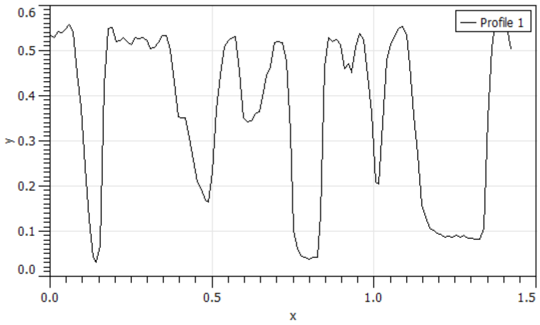

SEP-thf-casted-120

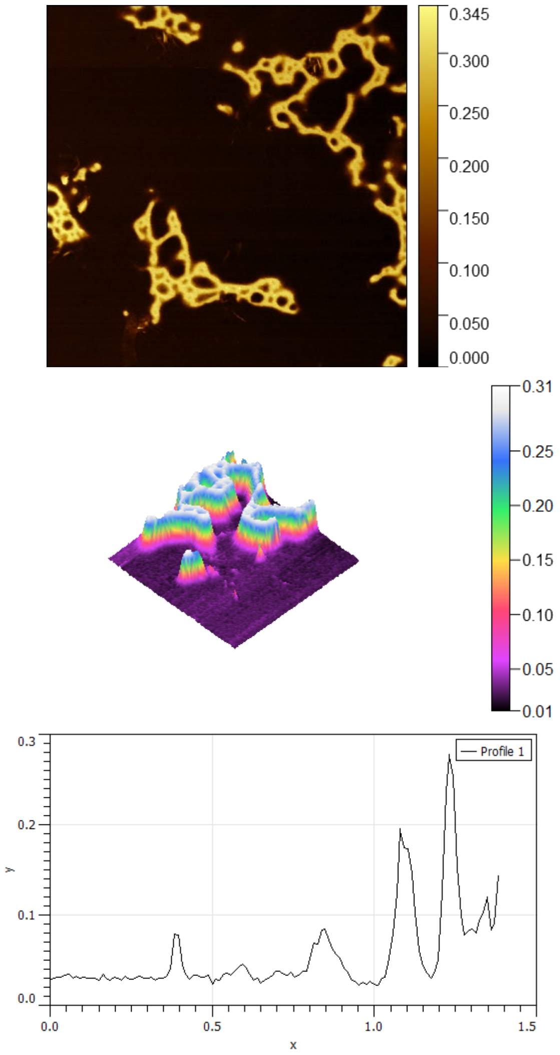

SEP-thf-spin casted-120

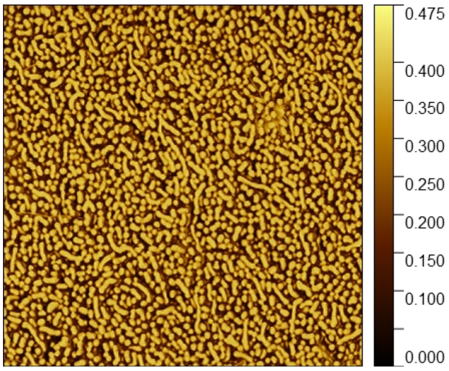

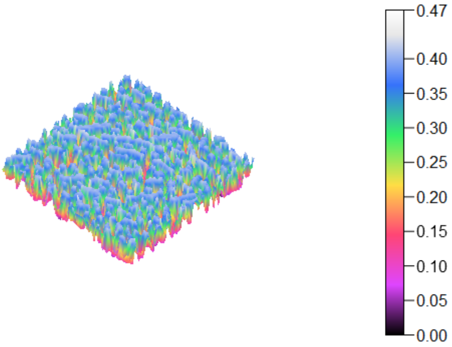

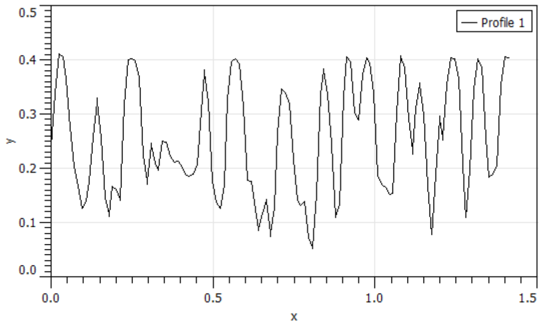

SI/sulf-cyclohexane-casted-RT

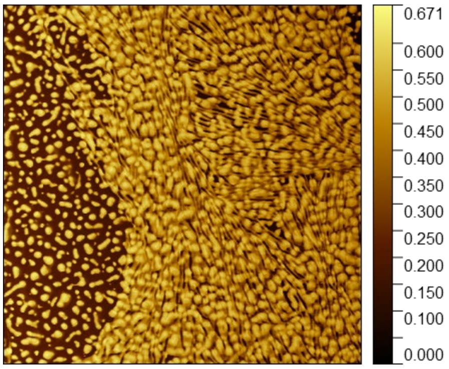

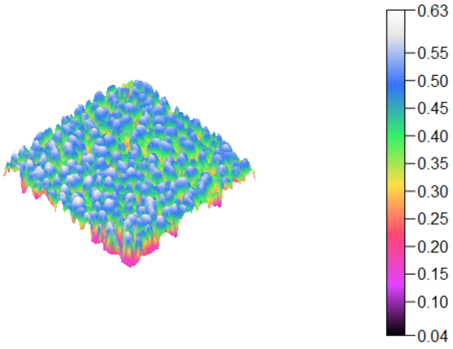

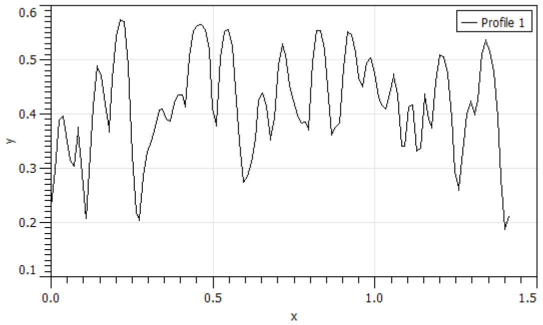

SI/sulf-cyclohexane-spin casted-RT

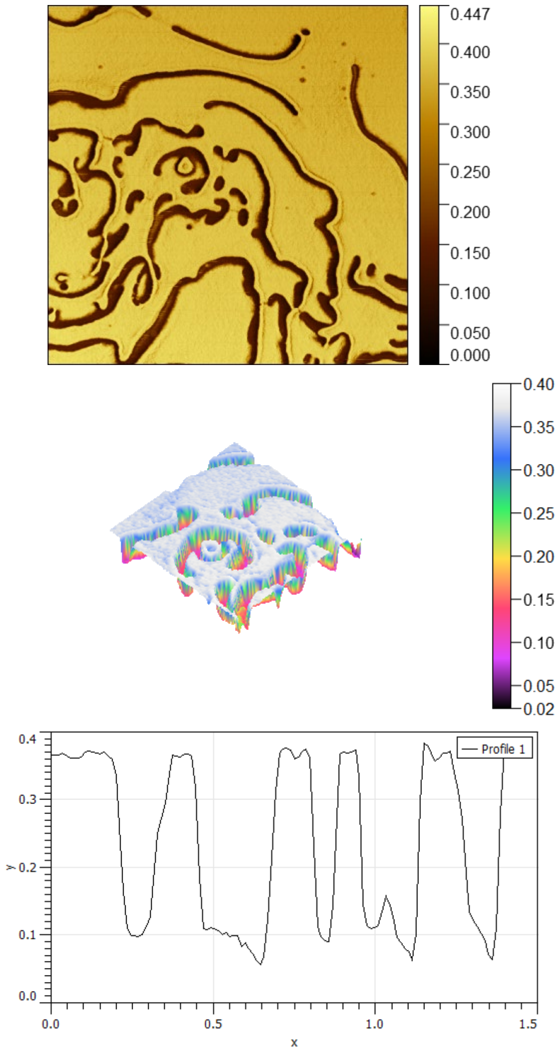

SI/sulf-toluene-casted-RT

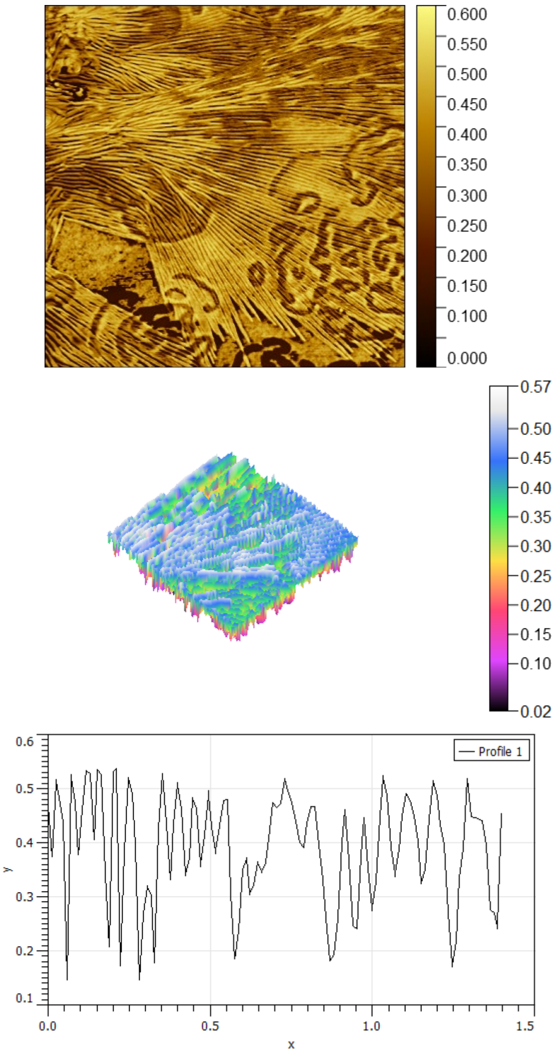

SI/sulf-toluene-spin casted-RT

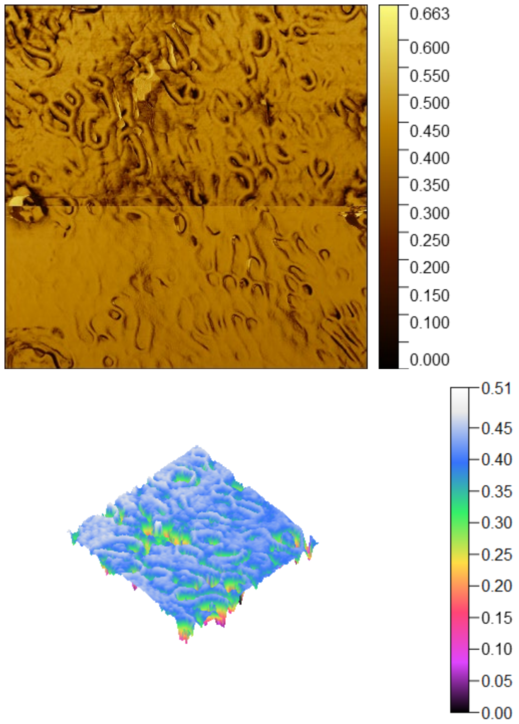

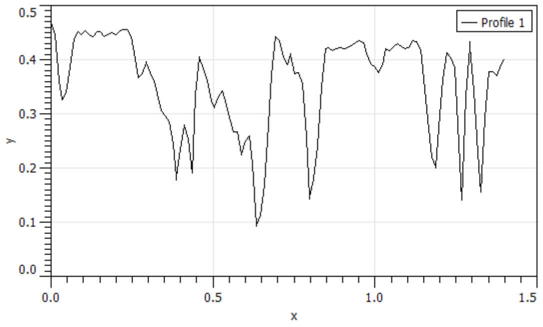

SI/sulf-thf-casted-RT

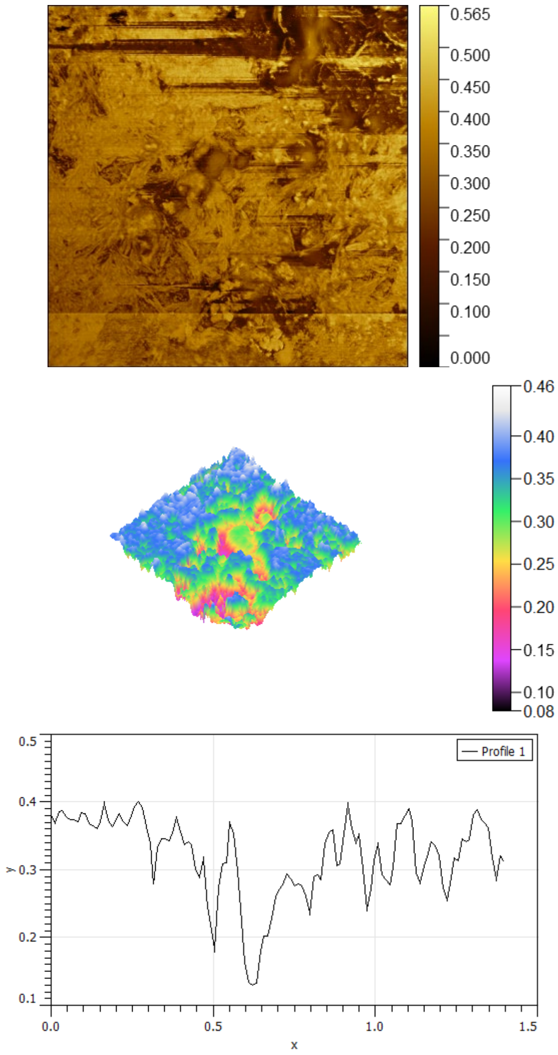

SI/sulf-thf-spin casted-RT

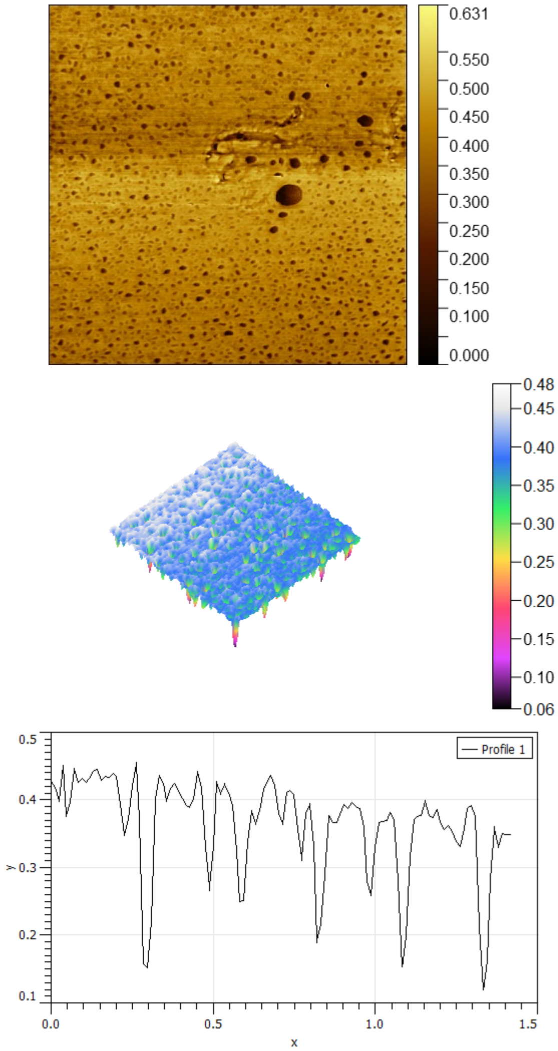

SI/sulf-cyclohexane-casted-80

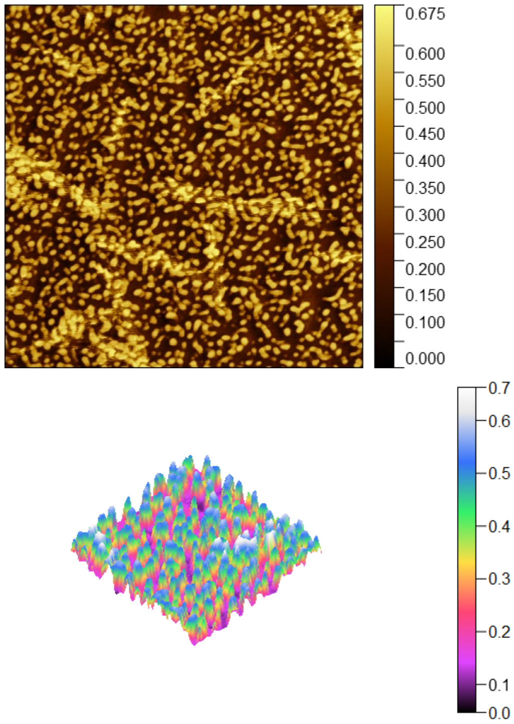

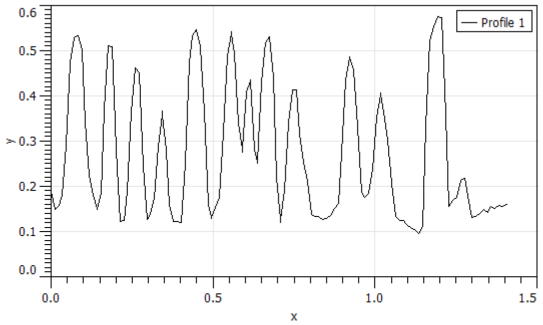

SI/sulf-cyclohexane-spin casted-80

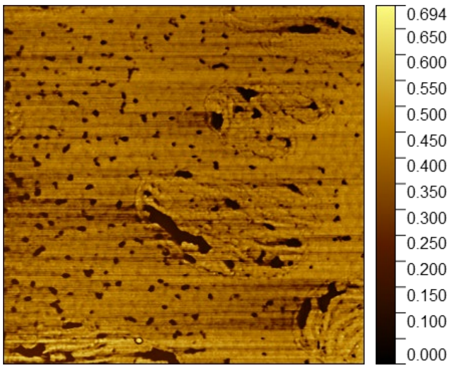

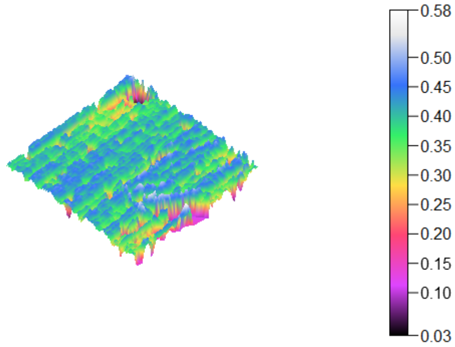

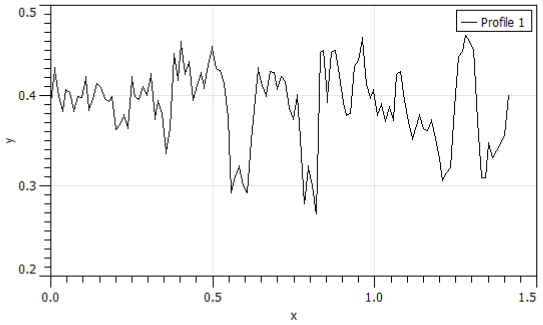

SI/sulf-toluene-casted-80

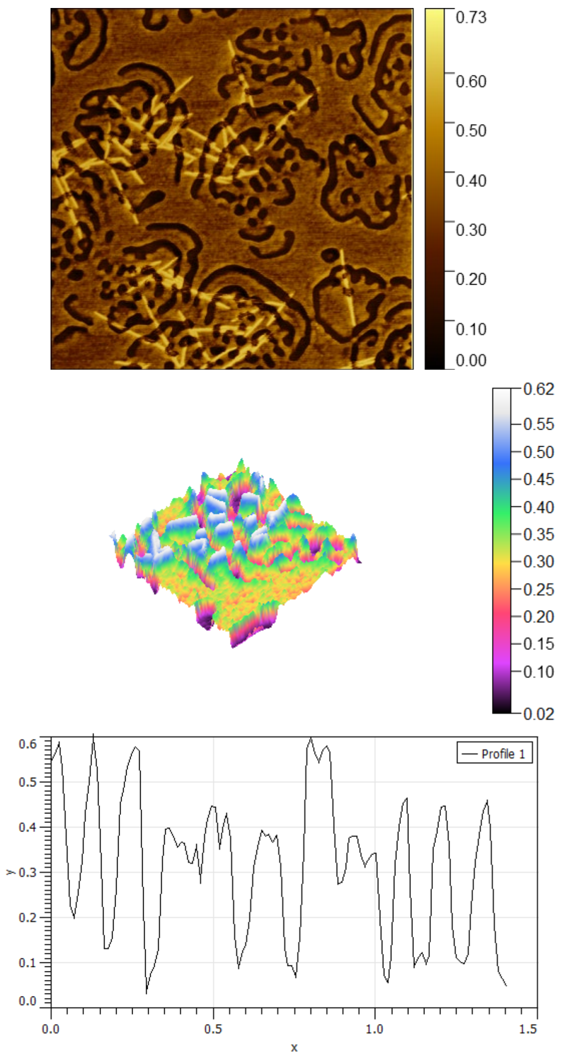

SI/sulf-toluene-spin casted-80

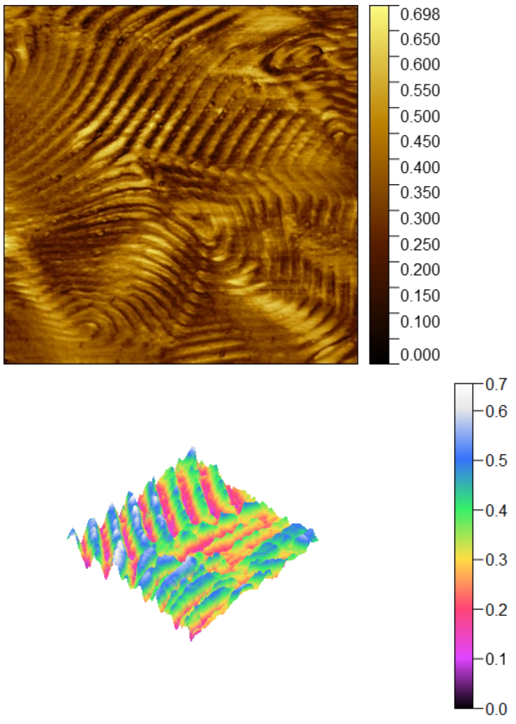

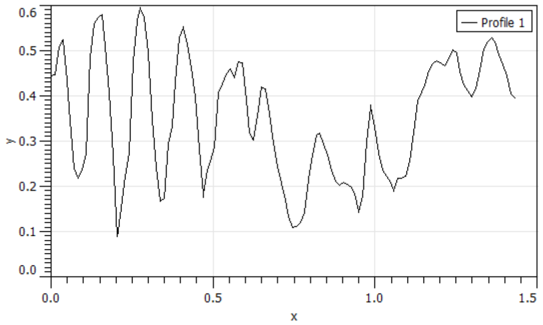

SI/sulf-thf-casted-80

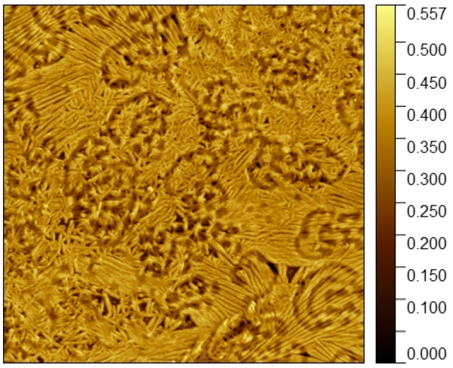

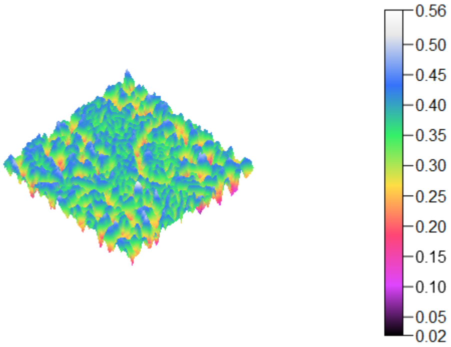

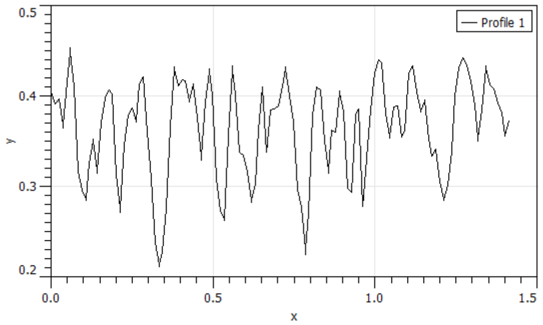

SI/sulf-thf-spin casted-80

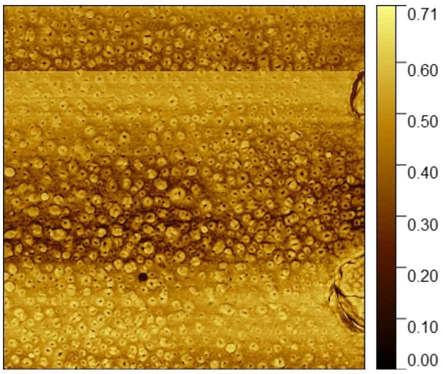

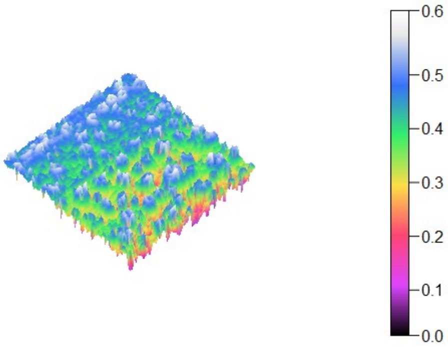

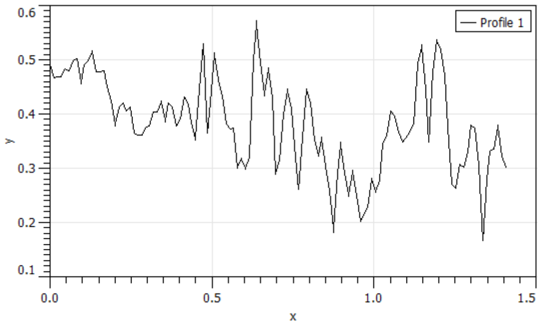

SI/sulf-cyclohexane-casted-100

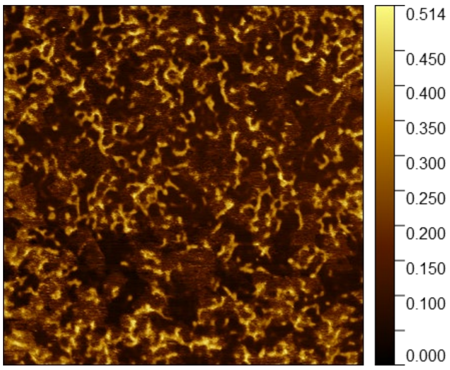

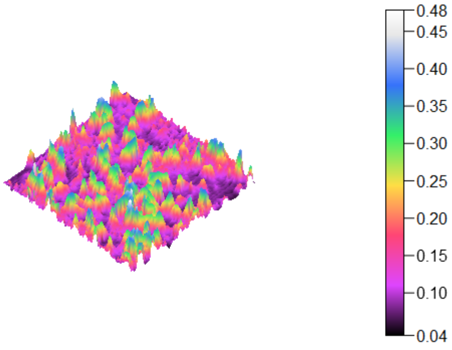

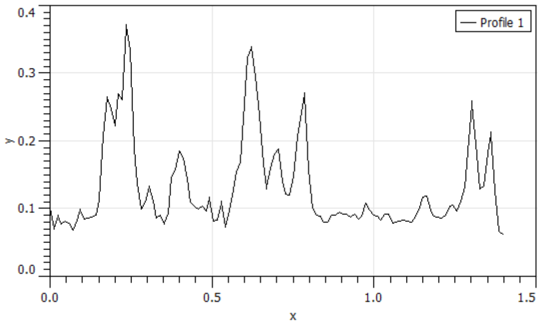

SI/sulf-cyclohexane-spin casted-100

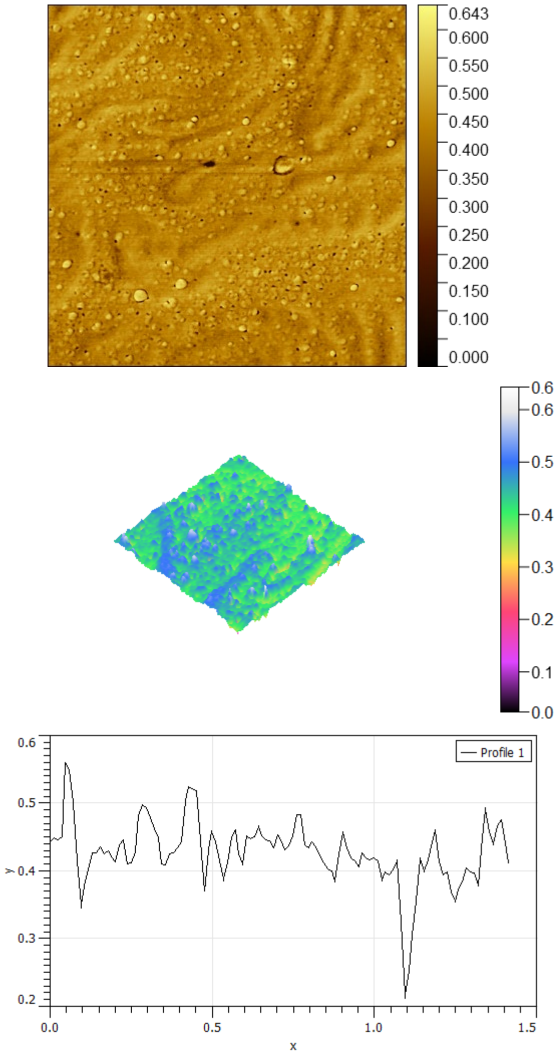

SI/sulf-toluene-casted-100

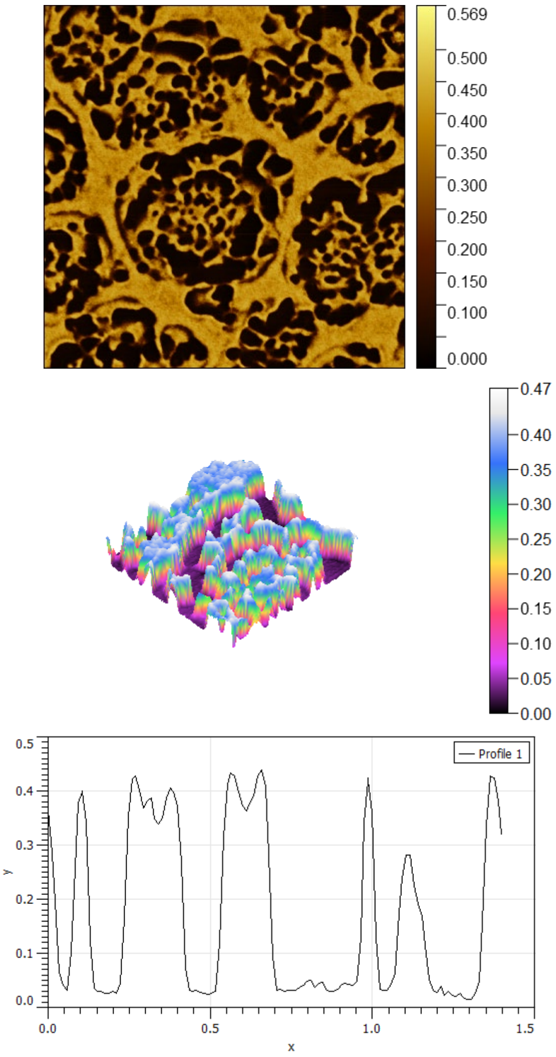

SI/sulf-toluene-spin casted-100

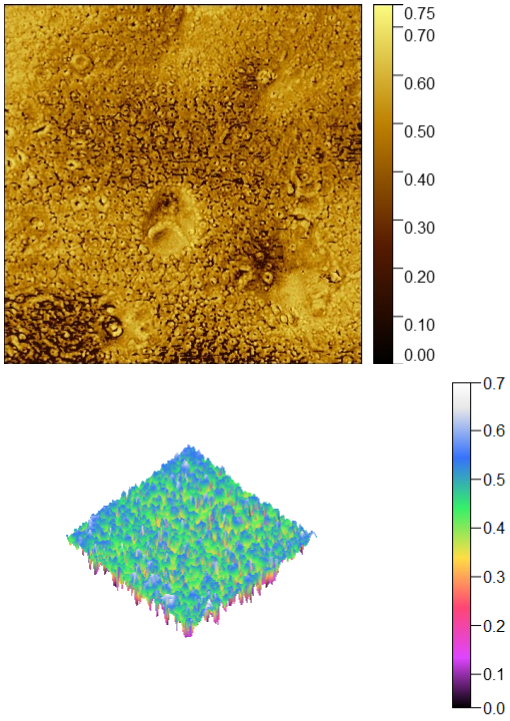

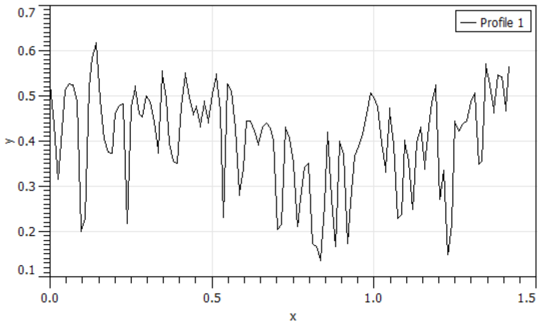

SI/sulf-thf-casted-100

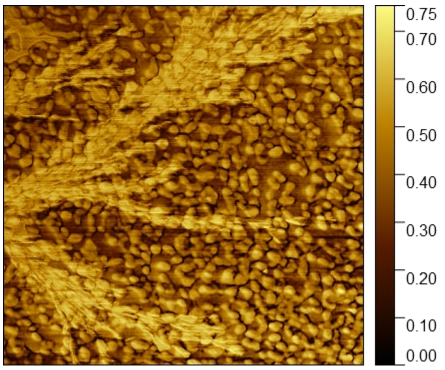

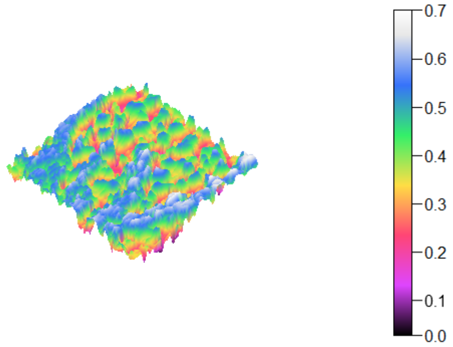

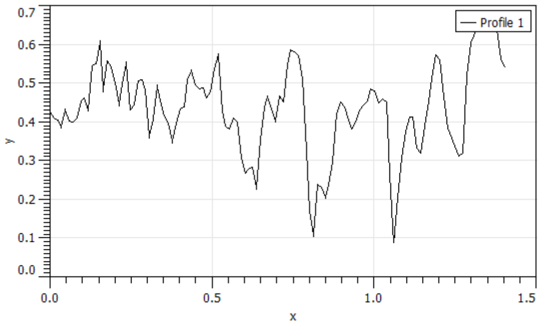

SI/sulf-thf-spin casted-100

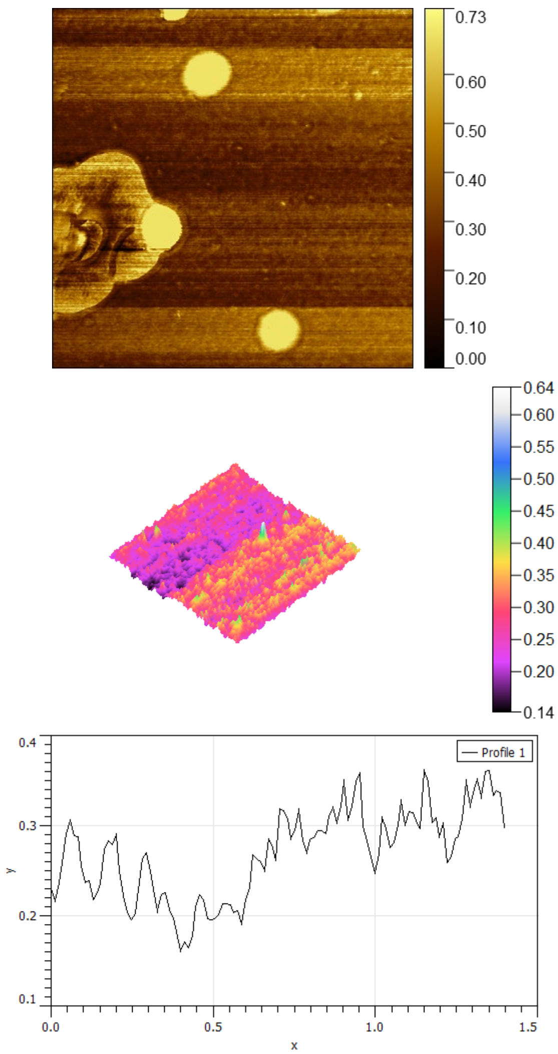

SI/sulf-cyclohexane-casted-120

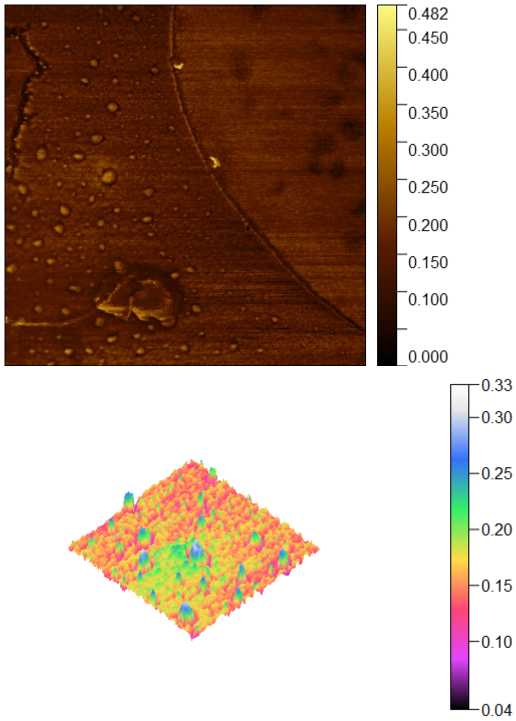

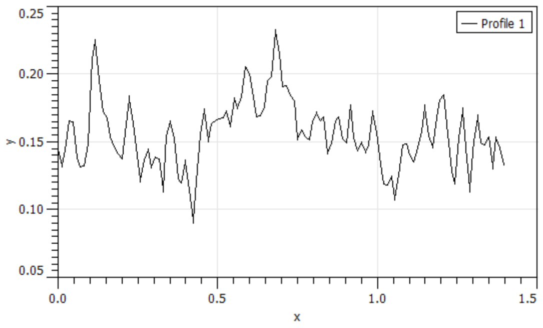

SI/sulf-cyclohexane-spin casted-120

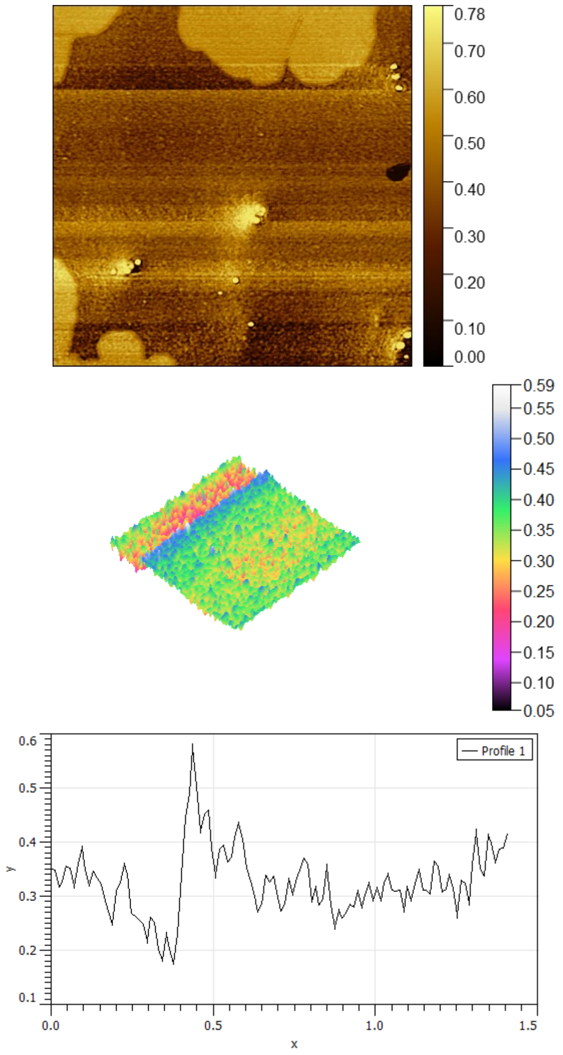

SI/sulf-toluene-casted-120

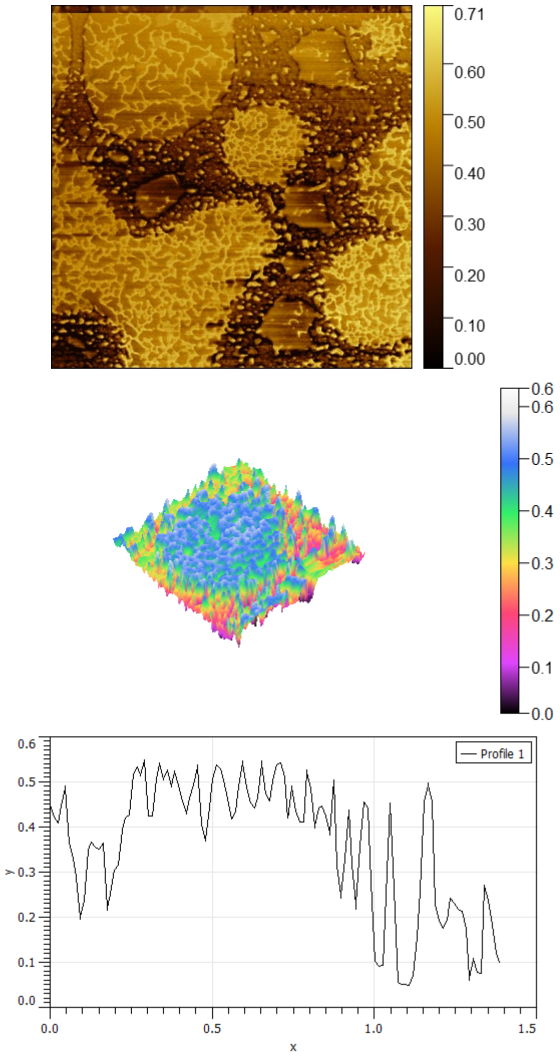

SI/sulf-toluene-spin casted-120

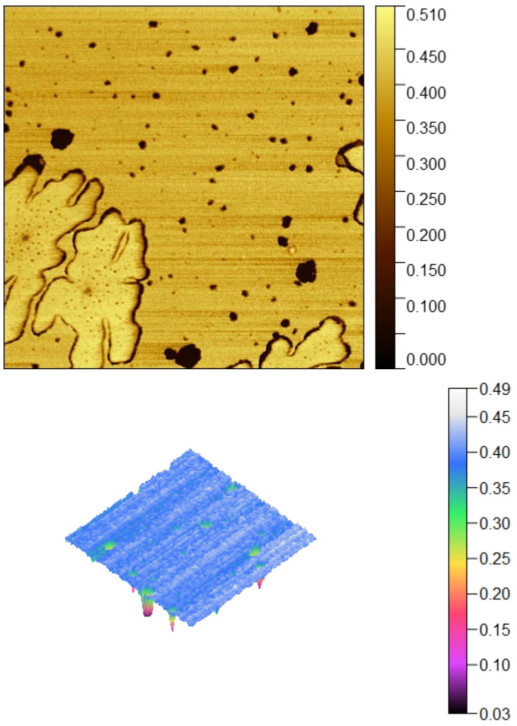

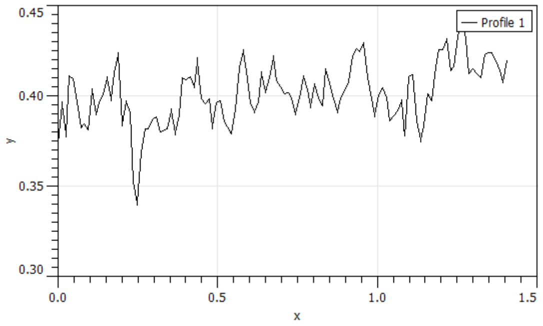

SI/sulf-thf-casted-120

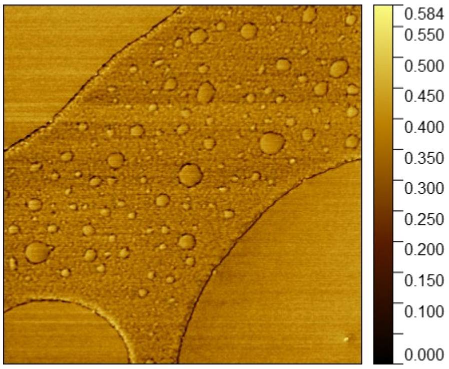

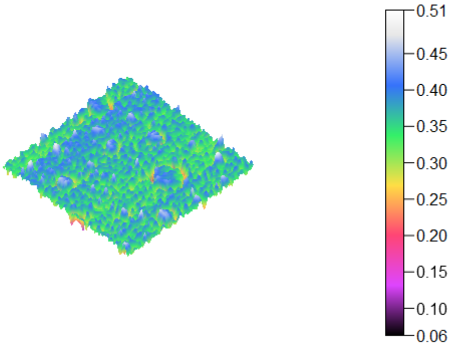

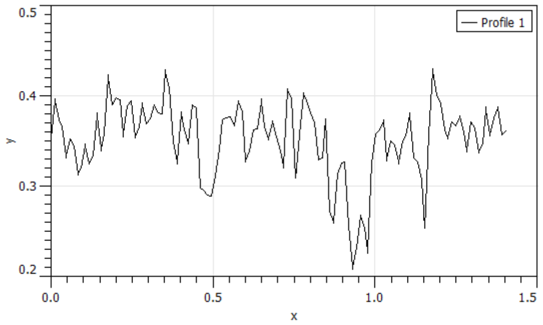

SI/sulf-thf-spin casted-120

Figure S2. All morphologies observed by AFM phase images (3μm x 3μm) with a 3D representation of a 1μm x 1μm and a profile of the morphology of 1.5μm diagonal.

Table S1. Characteristic values of roughness S_a, skewness S_k and domain sizes of bright and dark areas for all samples. The abbreviation for the samples is *sample-solvent-casting technique-annealing temperature*.

Sample	S_a	S_k	Domain size bright area (nm)	Domain size dark area (nm)

**SI-cycl-C-RT**	0.155	-0.28	16	11
**SI-cycl-SC-RT**	0.116	0.46	47	-
**SI-tol-C-RT**	0.068	-1.88	394	80
**SI-tol-SC-RT**	0.101	-1.07	-	59
**SI-thf-C-RT**	0.125	-0.65	-	104
**SI-thf-SC-RT**	0.166	0.20	44	61
**SI-cycl-C-80**	0.028	-	-	-
**SI-cycl-SC-80**	0.124	-0.03	71	-
**SI-tol-C-80**	0.071	-2.36	-	72
**SI-tol-SC-80**	0.117	0.03	428 (37)*	93
**SI-thf-C-80**	0.035	-	-	-
**SI-thf-SC-80**	0.058	-	-	-
**SI-cycl-C-100**	0.055	-	-	-
**SI-cycl-SC-100**	0.049	-	-	-
**SI-tol-C-100**	0.086	-	-	-
**SI-tol-SC-100**	0.086	-	-	-
**SI-thf-C-100**	0.048	-	-	-
**SI-thf-SC-100**	0.064	-1.11	56	27
**SI-cycl-C-120**	0.050	-	-	-
**SI-cycl-SC-120**	0.031	-	-	-
**SI-tol-C-120**	0.051	-	-	-
**SI-tol-SC-120**	0.038	-	-	-
**SI-thf-C-120**	0.043	-	-	-
**SI-thf-SC-120**	0.039	-	-	-
**SEP-cycl-C-RT**	0.157	-0.01	53	-
**SEP-cycl-SC-RT**	0.150	-0.20	73	-
**SEP-tol-C-RT**	0.094	0.10	63	90
**SEP-tol-SC-RT**	0.157	0.42	46	79
**SEP-thf-C-RT**	0.071	-1.86	44	61
**SEP-thf-SC-RT**	0.193	-0.01	41	55
**SEP-cycl-C-80**	0.081	-1.75	73	-
**SEP-cycl-SC-80**	0.069	-	-	-
**SEP-tol-C-80**	0.200	0.35	59	81
**SEP-tol-SC-80**	0.132	0.28	52	-
**SEP-thf-C-80**	0.154	0.31	81	130
**SEP-thf-SC-80**	0.184	-0.02	46	59
**SEP-cycl-C-100**	0.161	-0.92	80	-
**SEP-cycl-SC-100**	0.157	-0.49	-	71
**SEP-tol-C-100**	0.074	-	-	-
**SEP-tol-SC-100**	0.147	-0.10	57	90
**SEP-thf-C-100**	0.207	-0.10	50	82
**SEP-thf-SC-100**	0.132	0.05	54 (17)*	-
**SEP-cycl-C-120**	0.071	-1.15	64	63
**SEP-cycl-SC-120**	0.171	0.31	60	179
**SEP-tol-C-120**	0.172	-0.01	138	119
**SEP-tol-SC-120**	0.055	-	-	-
**SEP-thf-C-120**	0.170	-0.45	52	115
**SEP-thf-SC-120**	0.054	-	-	-

**SI/sulf-cycl-C-RT**	0.101	-0.29	52	-
**SI/sulf -cycl-SC-RT**	0.111	-0.35	71 (23)*	-
**SI/sulf -tol-C-RT**	0.073	-	-	-
**SI/sulf -tol-SC-RT**	0.095	-0.68	29	-
**SI/sulf -thf-C-RT**	0.051	-2.08	54	-
**SI/sulf -thf-SC-RT**	0.060	-	-	-
**SI/sulf -cycl-C-80**	0.037	-	-	-
**SI/sulf -cycl-SC-80**	0.140	0.37	49	-
**SI/sulf -tol-C-80**	0.057	-	-	-
**SI/sulf -tol-SC-80**	0.099	0.05	(42)*	72
**SI/sulf -thf-C-80**	0.072	0.08	82	79
**SI/sulf -thf-SC-80**	0.052	-0.94	35	-
**SI/sulf -cycl-C-100**	0.058	-0.42	84	22
**SI/sulf -cycl-SC-100**	0.068	1.10	39	-
**SI/sulf -tol-C-100**	0.031	-0.73	50	28
**SI/sulf -tol-SC-100**	0.151	0.05	48	-
**SI/sulf -thf-C-100**	0.086	-1.21	-	24
**SI/sulf -thf-SC-100**	0.105	-0.51	89	-
**SI/sulf -cycl-C-120**	0.089	-	-	-
**SI/sulf -cycl-SC-120**	0.050	-	-	-
**SI/sulf -tol-C-120**	0.082	-	-	-
**SI/sulf -tol-SC-120**	0.113	-	-	-
**SI/sulf -thf-C-120**	0.037	-	-	-
**SI/sulf -thf-SC-120**	0.031	-	-	-

*The value inside the parenthesis refers to the size of the crystaline domains.
